# Identification of Chimeric RNAs in Pig Skeletal Muscle and Transcriptomic Analysis of Chimeric RNA TNNI2-ACTA1 V1

**DOI:** 10.3389/fvets.2021.742593

**Published:** 2021-10-27

**Authors:** Dongyu Liu, Jiqiao Xia, Zewei Yang, Xuelian Zhao, Jiaxin Li, Wanjun Hao, Xiuqin Yang

**Affiliations:** College of Animal Sciences and Technology, Northeast Agricultural University, Harbin, China

**Keywords:** chimeric RNA, pig, skeletal muscle, growth, cell proliferation, transcriptomics

## Abstract

Chimeric RNA was considered a special marker of cancer. However, recent studies have demonstrated that chimeric RNAs also exist in non-cancerous cells and tissues. Here, we analyzed and predicted jointly 49 chimeric RNAs by Star-Fusion and FusionMap. One chimeric RNA, we named TNNI2-ACTA1, and its eight transcript variants were identified by reverse transcriptase–polymerase chain reaction. The overexpression of TNNI2-ACTA1 V1 inhibited the proliferation of porcine skeletal muscle satellite cells through down-regulating the mRNA expression levels of cell cycle–related genes cyclinD1. However, as parental genes, there is no such effect in the TNNI2 and ACTA1. To explore the underlying mechanism for this phenomenon, we used RNA-seq to profile the transcriptomes of PSCs with overexpression. Compared with the negative control group, 1,592 differentially expressed genes (DEGs) were upregulated and 1,077 DEGs downregulated in TNNI2 group; 1,226 DEGs were upregulated and 902 DEGs downregulated in ACTA1 group; and 13 DEGs were upregulated and 16 DEGs downregulated in TNNI2-ACTA1 V1 group, respectively. Compared with the parental gene groups, three specific genes were enriched in the TNNI2-ACTA1 V1 group (NCOA3, Radixin, and DDR2). These three genes may be the key to TNNI2-ACTA1 V1 regulating cell proliferation. Taken together, our study explores the role of chimeric RNAs in normal tissues. In addition, our study as the first research provides the foundation for the mechanism of chimeric RNAs regulating porcine skeletal muscle growth.

## Introduction

Chimeric RNAs are new RNAs formed by the fusion of two or more independent genes (parental genes) (1), which has an excellent application prospect and important research value in medicine (2–5). In past studies, Chimeric RNAs have been believed to be solely produced by gene fusions resulting from chromosomal rearrangement (6). However, there is currently sufficient evidence that chimeric RNAs can also be formed via various RNA splicing events including *cis*-splicing between adjacent genes (*cis*-SAGe) and *trans*-splicing (TS) (7).

The formation of chimeric RNA is an important biological phenomenon. The existence of chimeric RNAs has been found from lower animals to higher animals (8, 9), which attracted the attention of scientists gradually. With the development of a new generation of high-throughput sequencing technology, chimeric RNAs in mounting numbers have been found and identified. The implementation of the ENCODE studies revealed that ~65% of the genes in the human genome are involved in the formation of chimeric RNAs (10). Chimeric mRNAs fused by two previously separate genes located on different genomic loci may allow a limited number of genes to encode a substantially large number of mRNAs and proteins (11). They can change the function of the parental gene by connecting the domains of different genes, increasing the diversity and complexity of transcriptomics and proteomics. Chimeric RNAs are considered a genetic hallmark of many neoplasias because they can perturb the normal signal pathways or promote cancer cell growth (12). Recent studies have demonstrated that chimeric RNAs also exist in non-cancerous cells and tissues (13). Although chimeric RNA has shown important biological significances, such as in the field of medicine (5), its role in normal cells and tissues of animals is rarely reported.

Pigs are a crucial source of meat production worldwide and a potential medical model for human health issues (14). Skeletal muscle quantity and quality are regarded as the main indexes to measure meat quality (15). Skeletal muscle growth and development are a quite complex process, including muscle-derived stem cells to differentiate into muscle cells and monocytes, cell fusion into multinucleated myotubes, mature muscle fibers, and so on (16). Previous studies have reported that a series of factors regulated the proliferation and differentiation of muscle satellite cells, such as the signaling pathways, the transcription factors, and the epigenetic modifications (17). Although the process of skeletal muscle growth and development has been well-explored, the factors involved in the regulation of myogenesis are needed to be further explored.

In this study, Star-Fusion and FusionMap were chosen to analyze chimeric RNA in the six RNA-seq data sets. We use the Cell Counting Kit-8 (CCK-8) assay, 5-ethynyl-2′-deoxyuridine (EdU) staining, and flow cytometry to detect the effects of proliferation. Transcriptome analysis of porcine skeletal muscle satellite cells (PSCs) was conducted under the overexpression of TNNI2, ACTA1, and TNNI2-ACTA1 V1, and the results were compared with the negative control (NC) group using RNA sequencing technology (RNA-seq). Gene Ontology (GO) terms and the Kyoto Encyclopedia of Genes and Genomes (KEGG) pathway analysis were adopted to characterize the expression profiles in the PSCs of TNNI2, ACTA1, and TNNI2-ACTA1 V1. The aim of this study was to reveal the molecular mechanism underlying the difference in regulating the development of PSCs among TNNI2, ACTA1, and TNNI2-ACTA1 V1, which explore the role of chimeric RNAs in normal tissues.

## Materials and Methods

### Chimeric RNA Prediction

The RNA-seq data sets used in this study for chimeric RNA prediction come from our laboratory (18). In this study, two bioinformatics software, Star-Fusion and FusionMap, were used to jointly predict Chimeric RNAs. STAR-Fusion is a component of the Trinity Cancer Transcriptome Analysis Toolkit project that can leverage chimeric and predict fusions through discordant read alignments identified by the STAR aligner (19) (https://github.com/STAR-Fusion/STAR-Fusion). FusionMap can detect fusion events in both single- and paired-end data sets from either RNA-seq or DNA-seq studies and characterize fusion junctions at base-pair resolution (20) (http://www.arrayserver.com/wiki/index.php?title=Oshell). These two methods use different approaches, but generally include three main steps: (a) read alignment, (b) fusion candidate detection, and (c) false-positive elimination. They take Illumina RNA-seq data as input and generate lists of candidate fusion transcripts as output. The default settings were used to nominate fusion transcripts.

### Total RNA Extraction and Reverse Transcriptase–Polymerase Chain Reaction

Different primary tissues were first homogenized with mortar-and-pestle grinding in the presence of liquid N2. Total RNA of each sample was extracted by using TRIzol Reagent (Invitrogen Corporation, Carlsbad, CA, USA) following the manufacturer's instructions. The RNA concentration, RIN value, 28S/18S ratio, and the fragment length distribution were evaluated using an Agilent 2100 Bioanalyzer (Agilent Technologies Inc, USA). Total RNA was reverse transcribed to cDNA by using the PrimeScript™ RT Reagent Kit (Takara, Japan). The cDNA from total RNA was used as the template for the subsequent validation processes. The presence of fusions candidates generated by Star-Fusion and FusionMap algorithm from analyzed RNA-seq data sets was confirmed by reverse transcriptase–polymerase chain reaction (RT-PCR) (Supplementary Table 1). Following RT-PCR and gel electrophoresis, all purified bands were submitted for Sanger sequencing by the Beijing Genomics Institute.

### Expression Plasmid Constructions

A eukaryotic expression vector (pCMV-HA) carrying the TNNI2, ACTA1, and chimeric RNAs TNNI2-ACTA1 V1–V8 porcine gene was generated by cloning the coding sequence into the pCMV-HA vector. RNA samples from pig muscle were reverse-transcribed to cDNA, and full-length TNNI2, ACTA1, and chimeric RNA TNNI2-ACTA1 V1–V8 cDNA were amplified using primers in Supplementary Table 2.

### Cell Culture and Transfection

PSCs were purchased from MingZhou Biological Technology Limited Company (Ningbo, China). PSCs were cultured in Dulbecco modified eagle medium (DMEM)/F-12 (Gibco, Shanghai, China) supplemented with 10% fetal bovine serum (CLARK) and 1% penicillin/streptomycin (Solarbio, Beijing, China). The cell culture medium was changed every 2 days. PSCs were seeded in 6-well plates with the growth medium. For overexpression, according to the manufacturer's instructions, cells at 70–80% confluent in serum-free DMEM/F12 with gene expression vector or empty vector and Lipofectamine 2,000 reagent (Invitrogen, USA) for 6 h.

### CCK-8 Assay

Forty-eight h after transfection, the cells were dissociated by trypsin. The processed cells were placed in a 96-well plate at 5,000 cells per well. At least three biological replicates were examined per group. Ten microliters of CCK-8 solution was added to each well and incubated for 1 h. Finally, absorbance was read at 450 nm with a BIO-RAD iMark.

### EdU

Forty-eight h after transfection, the medium was removed, and cultured cells were carefully washed two times with phosphate-buffered saline (PBS). Appropriate concentration of EdU (Beyotime, Shanghai, People's Republic of China) working solution was used to incubate the cells for 2 h and fixed by 4% polyformaldehyde. Hoechst 33,342 was used to stain the nucleus. The experimental steps are strictly in accordance with the instructions.

### Cell Cycle

Cell cycle assays were performed at 48 h posttransfection. The cells were spun at 1,000 g for 5 min, and cell pellets were washed once in PBS at 4°C. One milliliter of 70% ethanol was added to each sample, and the sample was incubated at 4°C for 2 h. The cells were spun at 1,000 g for 5 min, and cell pellets were washed once in PBS at 4°C. The supernatant was removed, and 1 mL propidium iodide working solution was added to each tube to resuspend the pellets. The sampled was incubated for 30 min and protected from light at 37°C. Then, treated samples were analyzed using a flow cytometer. In order to detect the changes of cell cycle–related genes, real-time PCR was performed (Supplementary Table 3).

### RNA Preparation and Sequencing

Total RNA was extracted from three biological replicates of each sample using TRIzol reagent (Invitrogen, USA). The RNA concentration, RIN value, 28S/18S ratio, and the fragment length distribution were evaluated using an Agilent, 2100 Bioanalyzer (Agilent Technologies Inc, USA). The RNA samples were prepared for the construction of the cDNA library. All the standards and procedures were performed following the manufacturer's protocols. The library preparations were sequenced on an Illumina Hiseq 4,000 platform (Illumina, San Diego, CA, USA), and 100-bp paired-end reads were generated.

### RNA-Seq Data Set Analysis and Differentially Expressed Gene Analysis

Clean reads were obtained by removing reads containing adapter or poly-N and low-quality reads from raw reads. Clean reads were aligned against NCBI Genome *Sus scrofa* (Sscrofa 11.1) with HISAT2 v2.0.4 (available online at http://www.ccb.jhu.EdU/software/hisat) (default setting). Then, gene expression was estimated using RSEM v1.3.0 (available online at http://deweylab.github.io/RSEM/), and the FPKM (fragments per kilobase of transcript sequence per millions of base pairs sequenced) value was calculated. In order to identify differentially expressed genes (DEGs), normalized expression data were analyzed with DEseq2 (fold change ≥2.00, *p* ≤ 0.05) and PossionDis (fold change ≥2.00, false discovery rate ≤ 0.001). The DEGs were sorted by the enrichment of GO categories and KEGG database in the Database for Annotation, Visualization and Integrated Discovery (DAVID) Bioinformatics Resources (available online at http://david.abcc.ncifcrf.gov/).

### Quantitative Real-Time PCR

Quantitative real-time PCR (qRT-PCR) was used to validate the RNA-seq data sets. The primers for the 14 DEGs are shown in Supplementary Table 4. Total RNA was extracted as described previously, and reverse transcribed to cDNA using PrimeScript™ RT reagent Kit (Takara, Japan). qRT-PCR was performed using specific primers and SYBR Green Master Mix (BioTek, China) on a BioRad iQ5 system (Bio-Rad, Hercules, CA, USA). The relative expression values were normalized, with the GAPDH gene serving as an internal control. After amplification, the relative fold change of the DEGs was calculated through the 2^−ΔΔCt^ algorithm.

## Results

### Identification of Chimeric RNAs in Muscle

**Figure 1:**
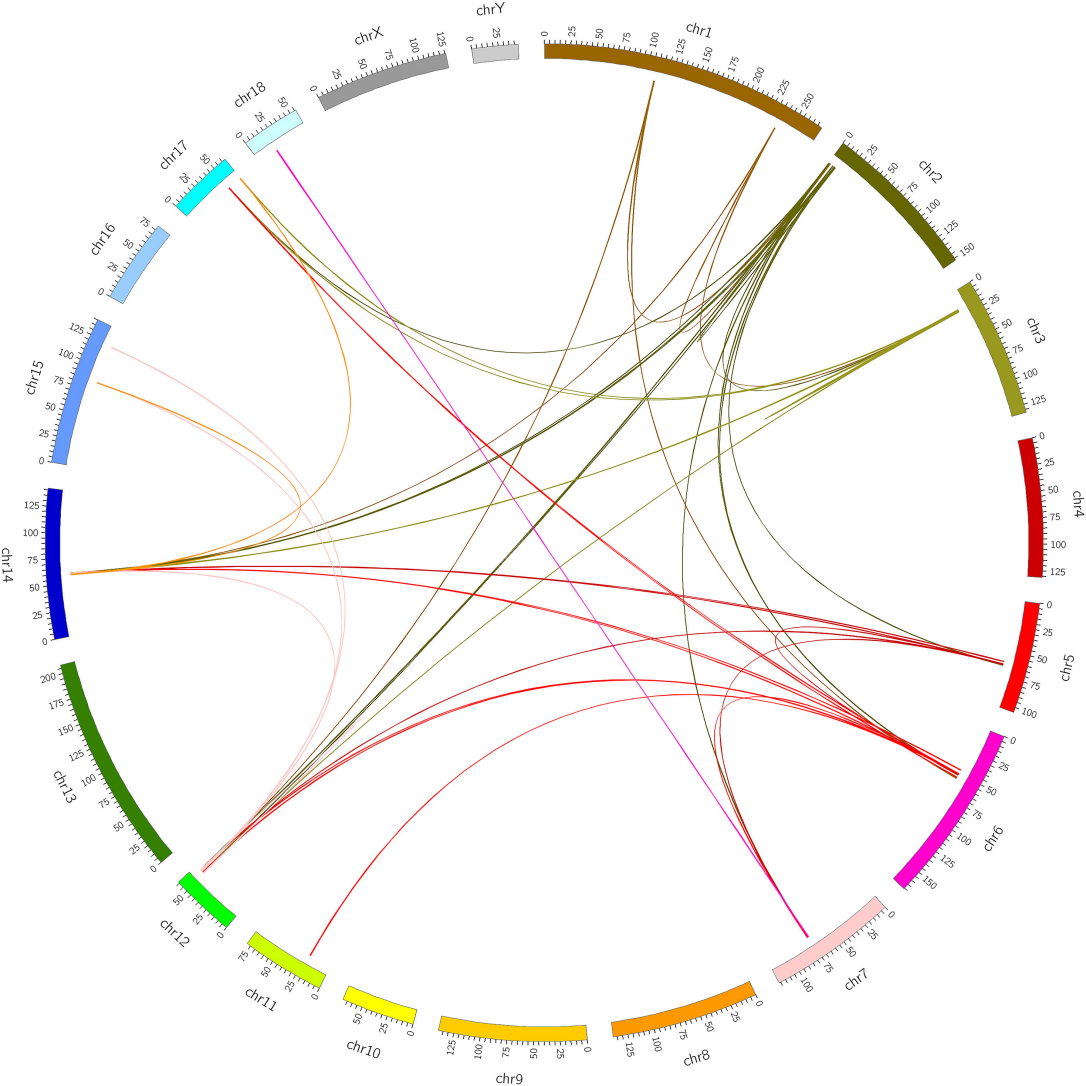
Identification of chimeric RNAs in pig muscle tissue. Chimeric RNAs were plotted on Circos plots. The fused transcripts are illustrated here as a line that connects two parental genes.

**Figure 2:**
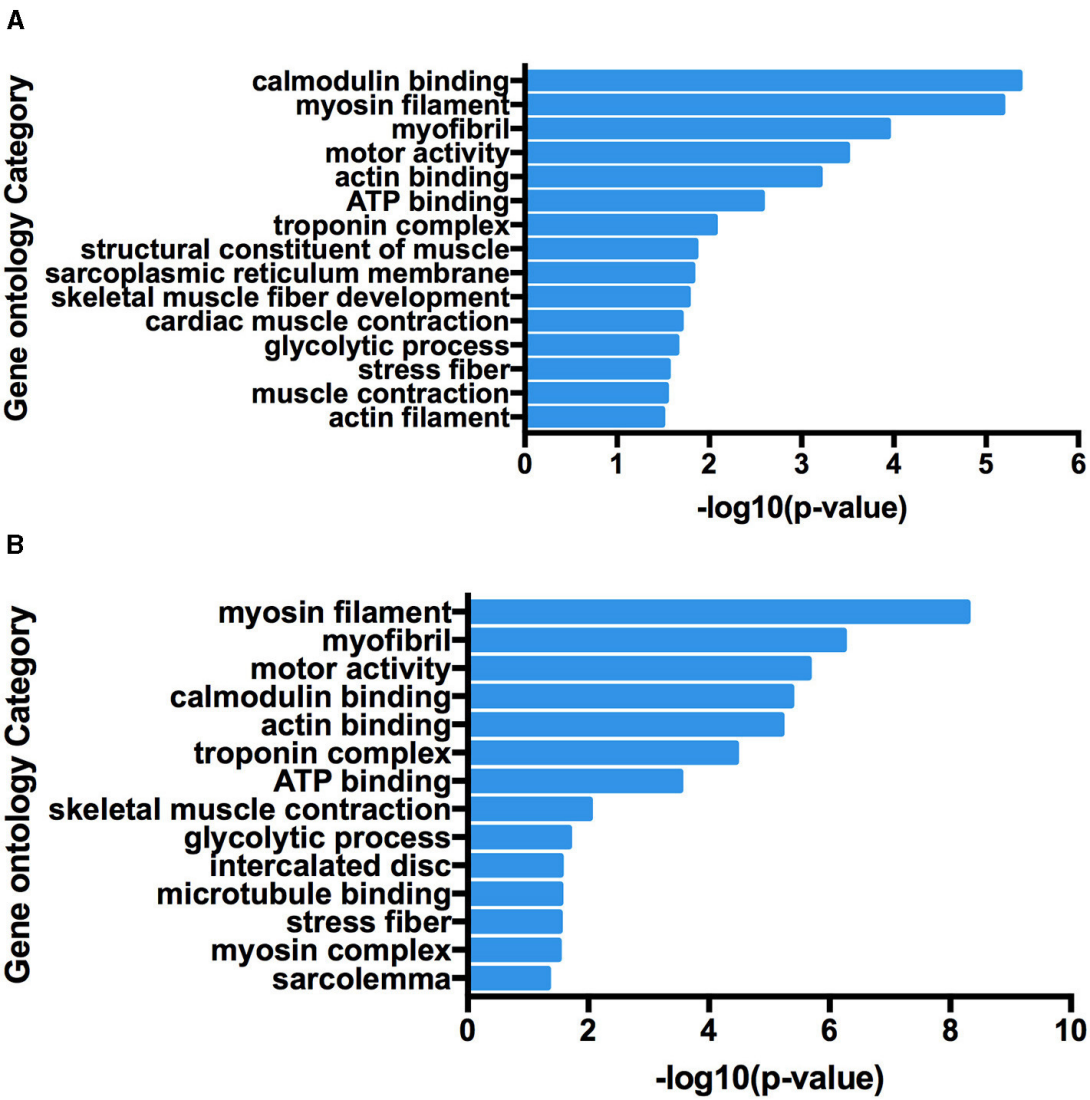
Functional map of differentially expressed genes enriched for GO terms. All categories were statistically significant (*p* < 0.05). **(A)** Gene Ontology terms enriched in 5′ parental genes involved in forming chimeric RNAs. **(B)** Gene Ontology terms enriched in 3′ parental genes involved in forming chimeric RNAs.

At present, many chimeric RNAs are found in normal human cell lines and tissues, with some data supporting their role in normal physiology (21). Global identification of fusion transcripts becomes possible with the help of next-generation sequencing (NGS) technology such as RNA-seq. In this study, in order to predict chimeric RNAs during muscle development, we analyzed three RNA-seq data sets of longissimus dorsi and three RNA-seq data sets of biceps femoris from Min pig. The bioinformatics software, Star-Fusion and FusionMap, were used to expose total of 49 chimeric RNAs (Supplementary Table 5). We then categorized these chimeras in order to explore their potential functions and mechanisms. Chimeric RNAs were classified according to the chromosomal location of their parental genes: parental genes located on different chromosomes (interchromosomal) and other fusions with parental genes on the same chromosome (intrachromosomal). The proportions of the two types of chimeric RNAs in this study were 88 and 12%, respectively. The landscape of the chimeras was illustrated using Circos plots (Figure 1). We examined GO terms for the parental genes involved in chimeric RNAs. The annotated results were classified into three parts: biological process, cellular component, and molecular function. GO enrichment revealed that there were 15 GO terms enriched in 5′ parental genes (*p* < 0.05) and 14 GO terms enriched in 3′ parental genes (*p* < 0.05). In both 5′ and 3′ parental genes, calmodulin-binding, myosin filament and myofibril were predominant (Figure 2).

### Validations of Selected Chimeric RNAs

**Figure 3:**
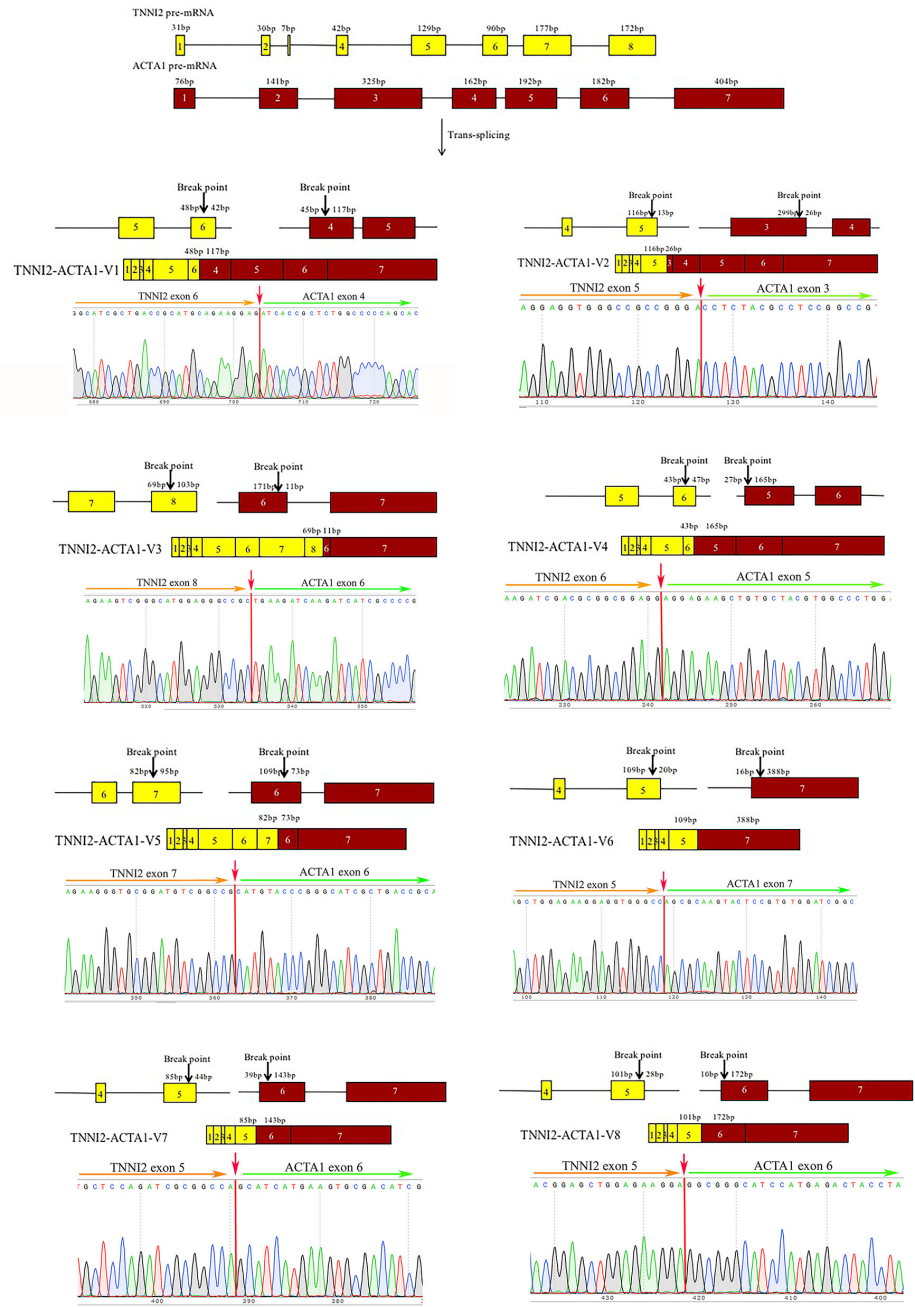
Structure and Sanger sequencing validation of chimeric RNA TNNI2-ACTA1 transcript variants V1–V8. The red line marks the fusion junction site. The yellow boxes indicate exons of TNNI2. The red boxes indicate exons of ACTA1. Yellow and green arrows indicate the direction of transcription.

To validate the reliability of the group of identified chimeric RNAs, four chimeric RNAs were selected for RT-PCR verification (ALDOA-ACTA1, MYH1-ACTA1, TNNT3-ACTA1, and TNNI2-ACTA1). The primers were designed to span the fusion junction of chimeric RNAs. Eight transcripts of chimeric RNA TNNI2-ACTA1 were successfully amplified by RT-PCR, and their sequences were confirmed by Sanger sequencing (Figure 3). We named these transcript variants V1–V8 (Table 1), respectively. Traditionally, gene fusions were thought to be generated solely by chromosomal rearrangements (12). However, recent discoveries of TS and *cis*-splicing events between neighboring genes suggest that there are other mechanisms to generate chimeric fusion RNAs without corresponding changes in DNA. According to the analysis results, TNNI2-ACTA1 transcript variants V1–V8 seem to result from TS between precursor mRNAs transcribed from the two intact genes TNNI2 and ACTA1.

**Table 1:** Specific information of chimeric RNA TNNI2-ACTA1transcript variants V1–V8.

**Name**	**GenBank**	**CDS length (bp)**	**5^′^ Gene**	**5^′^ Breakpoint**	**5^′^ BreakDinuc**	**3^′^ Gene**	**3^′^ Breakpoint**	**3^′^ BreakDinuc**
V1	MZ275226	870	TNNI2	Exon6	AG	ACTA1	Exon4	AT
V2	MZ275227	819		Exon5	GA		Exon3	CC
V3	MZ275228	525		Exon8	GC		Exon6	TG
V4	MZ275229	720		Exon6	GG		Exon5	AG
V5	MZ275230	423		Exon7	CG		Exon6	CA
V6	MZ275231	234		Exon5	CC		Exon7	AG
V7	MZ275232	369		Exon5	CA		Exon6	GC
V8	MZ275233	414		Exon5	GA		Exon6	GG

### Cell Viability

**Figure 4:**
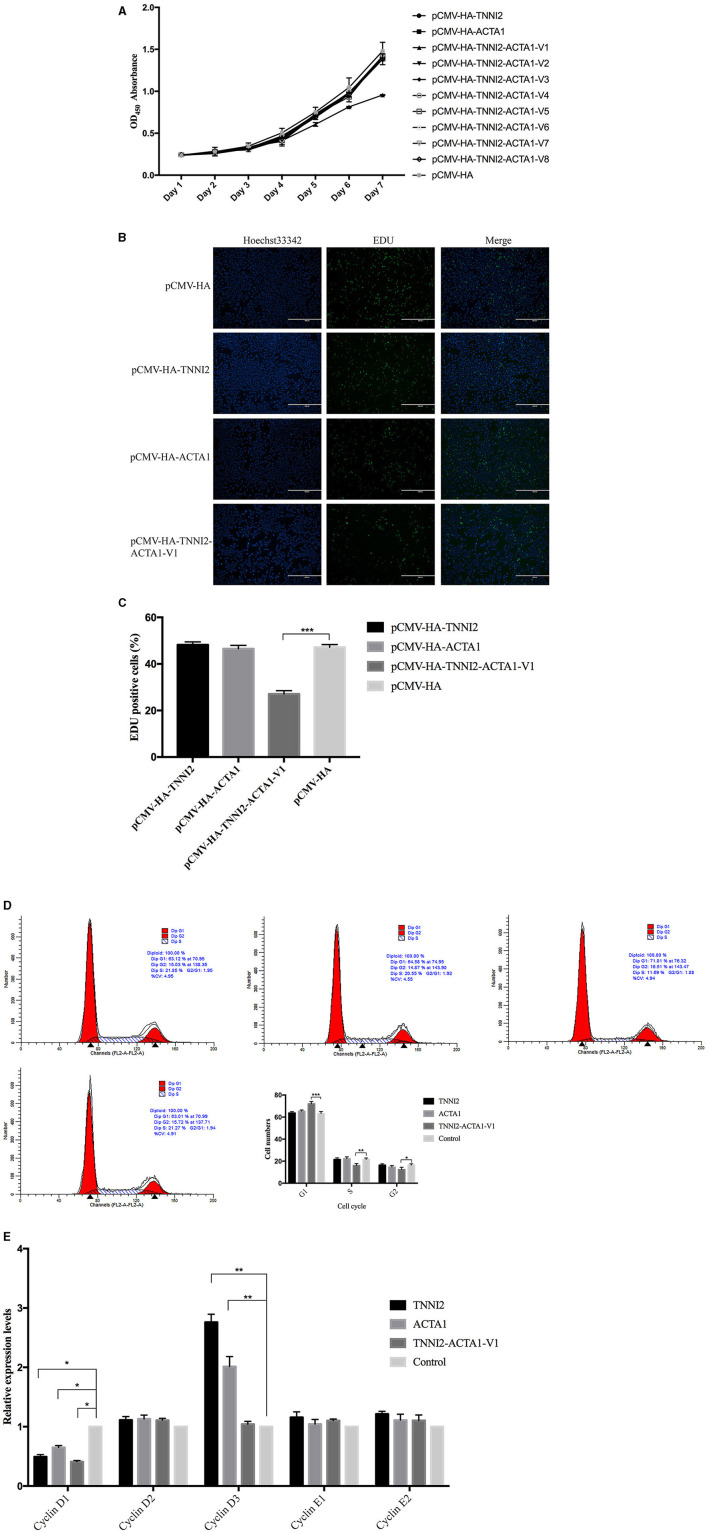
Effects of target fragments on cell proliferation of PSCs. **(A)** The results of the CCK-8 experiment to detect cell proliferation in 7 days. **(B)** EdU staining results of PSCs in different groups at 48 h posttransfection. **(C)** The relative percentage of EdU-positive cells in images of related groups is shown. **(D)** PSCs were transfected with target fragments overexpression plasmids during proliferation. The cells were harvested, and samples were analyzed by flow cytometry at 48 h posttransfection. **(E)** Detection of the variation of related gene expression of G1/S phase by real-time PCR. *p* < 0.05 was considered statistically significant. *p < 0.05, **p < 0.01, ***p < 0.001.

In order to explore the effects of TNNI2-ACTA1 transcript variants and their parental genes on the proliferation of PSCs, overexpression vectors pCMV-HA-TNNI2-ACTA1 V1–V8, pCMV-HA-TNNI2, and pCMV-HA-ACTA1 were constructed, respectively. The CCK-8 method was used to detect the effect of transfection of plasmid vector on PSC viability. As shown in Figure 4A, significant difference was observed in the cell viability after the cells were transfected with pCMV-HA-TNNI2-ACTA1 V1. Cell viability decreased significantly after the fifth day (*p* < 0.05) in the pCMV-HA-TNNI2-ACTA1 V1 group, and there are no significant effects in other groups. The CCK-8 assay indirectly showed that TNNI2-ACTA1 V1 can inhibit PSC proliferation.

### EdU Staining

We then analyzed the cell proliferation by EdU staining assay to confirm that TNNI2-ACTA1 V1 can inhibit PSC proliferation. The nuclei of all PSCs were stained with blue (Hoechst 33,342), and the nuclei of PSCs with high DNA replication activities (EdU-positive cells) were stained with green simultaneously.

The results revealed significantly fewer EdU-positive cells after transfection TNNI2-ACTA1 V1, compared with the control. EdU assay results showed that the number of proliferating PSCs in the TNNI2-ACTA1 V1 group was decreased (*p* < 0.001), compared with the NC group (Figures 4B,C). TNNI2-ACTA1 V2–V8, TNNI2, and ACTA1 groups have no obvious effect observed in the EdU staining. The EdU staining results showed that overexpression of TNNI2-ACTA1 V1 can inhibit PSC proliferation.

### Cell Cycle

To further determine the underlying mechanisms of TNNI2-ACTA1 V1 inhibition of the growth of PSCs, we examined the effects of TNNI2-ACTA1 V1 on the PSCs cell cycle. After transfection with plasmid pCMV-HA-TNNI2-ACTA1 V1 for 48 h, the proportion of PSCs in G1 phase most significantly increased from 63.2 to 72.08% (*p* < 0.001); the proportion in S phase extremely significantly decreased from 21.52 to 16.05% (*p* < 0.01); the proportion in G2 phase significantly decreased from 16.71 to 12.39% (*p* < 0.05) (Figure 4D). No significant differences in the number of cells in cell cycle were observed following transfection with plasmids pCMV-HA-TNNI2 and pCMV-HA-ACTA1, respectively. These results suggested that TNNI2-ACTA1 V1 induced cell cycle arrest at the G1 phase.

To gain insight into the mechanisms that TNNI2-ACTA1 V1 prevents the PSCs growth, we assessed the changes in mRNA expression of key proliferation-related genes in G1 phase (cyclinD1, cyclinD2, cyclinD3, cyclinE1, and cyclinE2). Our data showed the expression level of cyclinD1 was significantly decreased in the TNNI2-ACTA1 V1 group, TNNI2 group, and ACTA1 group (*p* < 0.05). Interestingly, the expression level of cyclinD3 was extremely significantly increased in both the TNNI2 group and ACTA1 group (*p* < 0.01). In addition, expression levels of cyclinD2, cyclinE1, and cyclinE2 did not change significantly (Figure 4E).

### RNA Sequencing Data Mapping and Annotation

In total, 12 cDNA libraries from four groups (TNNI2, ACTA1, TNNI2-ACTA1 V1, NC; three replications for each group) were sequenced, which yielded 286.91 million 100-bp paired-end clean reads in total, average from 23.91 million for each sample (Supplementary Table 6). Among the clean reads, more than 98.20% had quality scores at the Q20 level, and on average, approximately 91.13% of clean reads were mapped to the reference genome (Sscrofa 11.1).

After assembling for each sample, Cuffdiff package in Cufflinks was used to calculate the expected number of FPKM of the three groups for each gene according to the Sscrofa 11.1 reference genome annotation. In order to more intuitively display the number of genes in each sample in different FPKM intervals, we have performed statistics on the number of genes in the three cases of FPKM (FPKM ≤ 1, FPKM 1–10, FPKM ≥10) (Supplementary Figure 1A). The FPKM density of 12 data sets displayed similar distribution (Supplementary Figure 1B).

### Verification of the Expression Level of 14 DEGs by qRT-PCR

**Figure 5:**
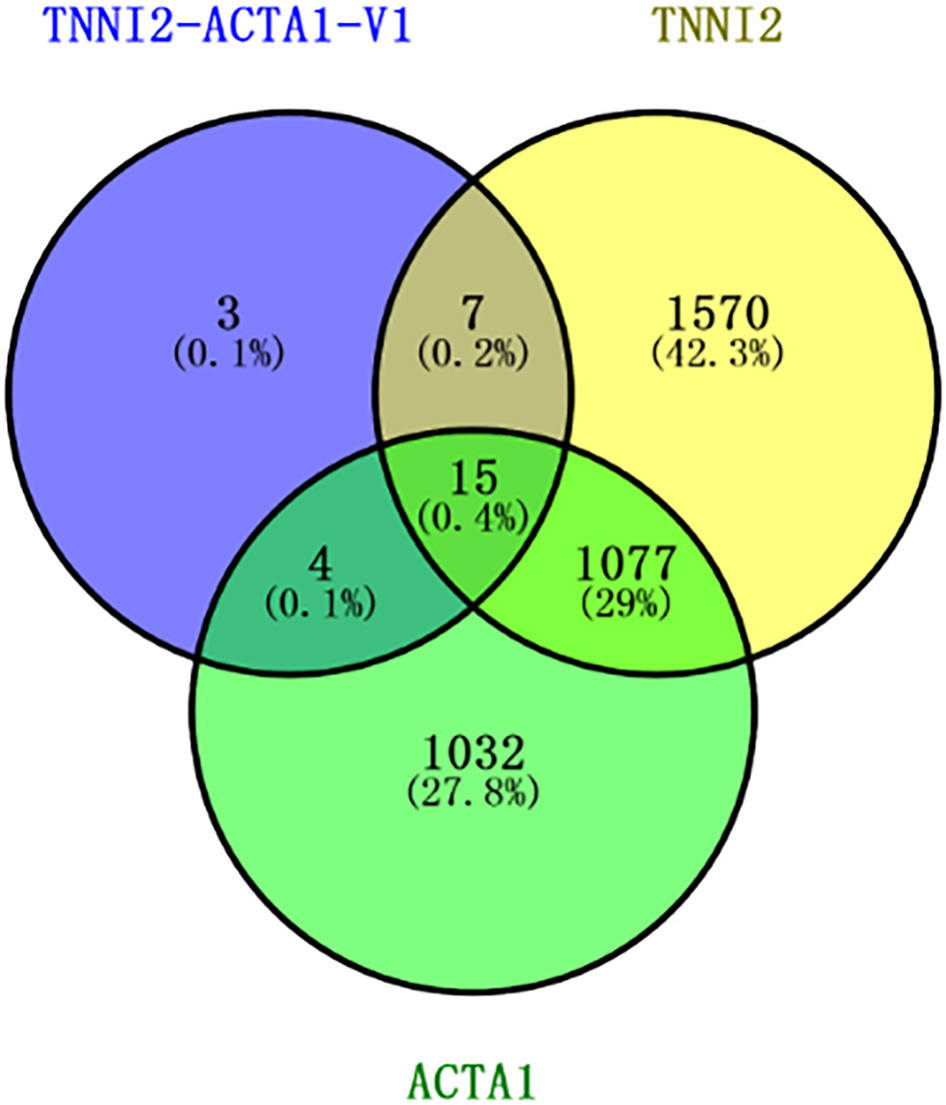
Venn diagram showing the number of specific DEGs genes among these three groups. The blue circle represents the TNNI2-ACTA1 V1 group. The yellow circle represents the TNNI2 group. The green circle represents the ACTA1 group.

To identify the DEGs in response to posttransfection in PSCs, all the gene numbers were homogenized by an algorithm of reads per kb per million reads, and then DEGs were generated by horizontally compared among TNNI2 vs. NC, ACTA1 vs. NC, and TNNI2-ACTA1 V1 vs. NC. Compared with NC, 1,592 DEGs were up-regulated and 1,077 DEGs down-regulated in TNNI2 group (*p* < 0.05) (Supplementary Figure 2A). In group ACTA1, compared with NC, 1,226 DEGs were up-regulated and 902 DEGs down-regulated (*p* < 0.05) (Supplementary Figure 2B). Thirteen DEGs were up-regulated and 16 DEGs down-regulated in TNNI2-ACTA1 V1 group, compared with NC (*p* < 0.05) (Supplementary Figure 2C). Compared with groups TNNI2 and ACTA1, the number of DEGs in TNNI2-ACTA1 V1 group is less. Our experiment results showed that TNNI2 and ACTA1 as the parental genes cannot inhibit PSC proliferation. We believe that the DEGs enriched specifically in TNNI2-ACTA1 V1 group is the key. As shown in Figure 5, three specific DEGs were found in group TNNI2-ACTA1 V1 (NCOA3, Radixin, and DDR2) compared with groups TNNI2 and ACTA1.

**Figure 6:**
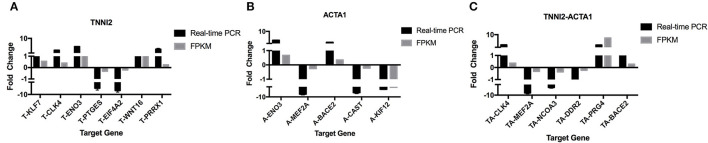
Real-time PCR validation of the DEGs analyzed by RNA-seq. The *x*-axis represents genes. The *y*-axis shows the relative expression levels. **(A)** Seven genes that were identified as DEGs in group TNNI2. **(B)** Five genes that were identified as DEGs in group ACTA1. **(C)** Six genes that were identified as DEGs in group TNNI2-ACTA1 V1.

**Figure 7:**
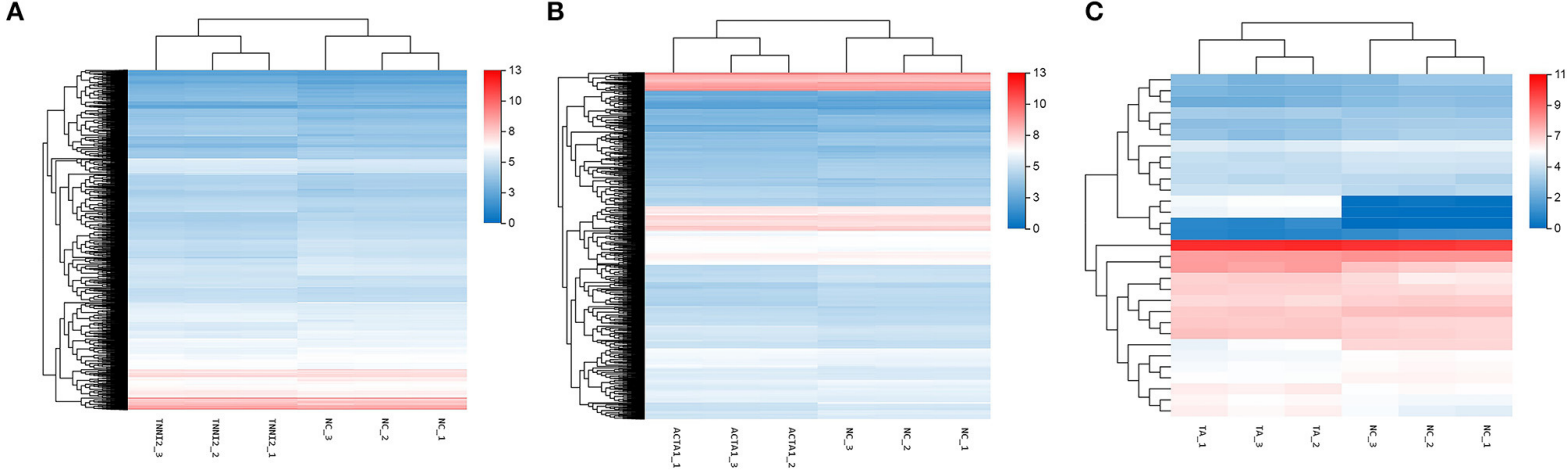
The expression level of DEGs displayed with heat map. The *x*-axis represents samples. The *y*-axis represents DEGs. The color depth represents –log10 (FPKM). The red means high expression level; the blue means low expression level. **(A)** DEGs of group TNNI2. **(B)** DEGs of group ACTA1. **(C)** DEGs of group TNNI2-ACTA1 V1.

To validate the accuracy of the RNA-seq results, 14 DEGs (NCOA3, DDR2, Radixin, KLF7, CLK4, PTGES, EIF4A2, MEF2A, CAST, PRRX1, WNT16, ENO3, BACE2, and PRG4) and a housekeeping gene GAPDH were selected for qRT-PCR validation. Among these genes, seven genes were selected for group TNNI2, five genes were for group ACTA1, and six genes were for group TNNI2-ACTA1 V1. The results showed that the expression patterns of these genes as measured using real-time PCR were consistent with those obtained via RNA-seq (Figure 6). The above results indicate that the DEG identified by RNA-seq was reliable. The heat maps of the three groups showed that the three clusters related to the muscle have three different gene expression patterns (Figure 7).

### Functional Enrichment and Pathway Analysis of DEGs

**Figure 8:**
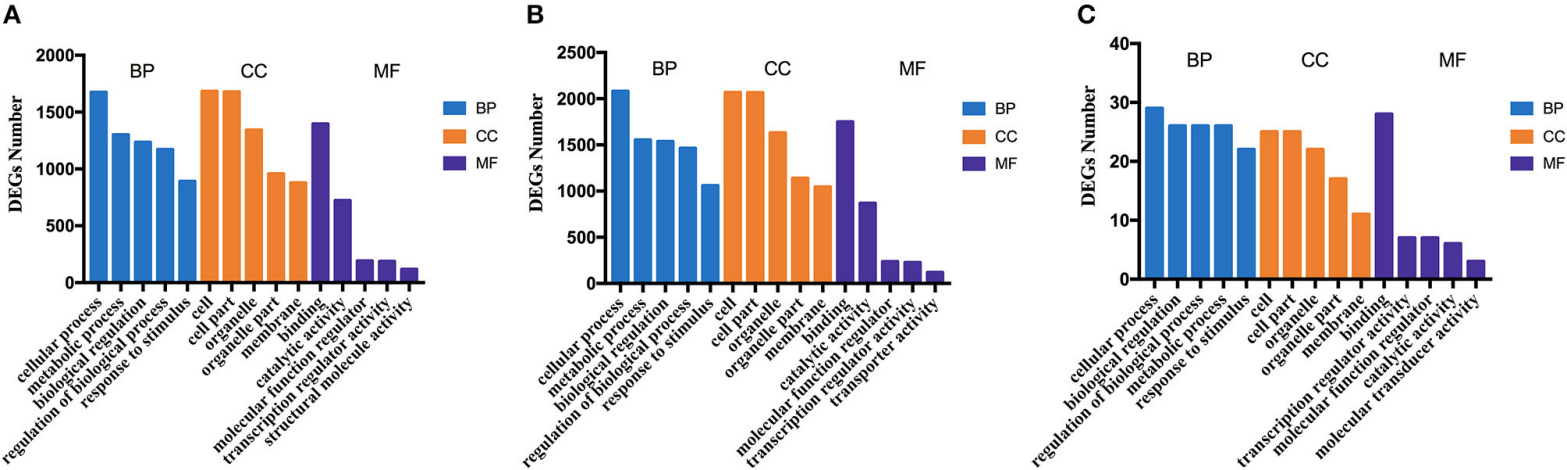
The column diagrams for Gene Ontology (GO) analysis of DEGs. The *x*-axis represents the functions of GO analysis. The *y*-axis represents the numbers of DEGs. **(A)** DEGs of group TNNI2. **(B)** DEGs of group ACTA1. **(C)** DEGs of group TNNI2-ACTA1 V1.

The potential functions and metabolic pathways of the identified DEGs were analyzed by GO and KEGG enrichment analysis. The GO analyses of DEGs were divided into three categories: biological process, cell component, and molecular function. The top five of each part for these three groups are shown in Figure 8. According to the function, pathways are divided into six classifications: cellular processes, environmental information processing, human diseases, genetic information processing, metabolism, and organismal systems (Supplementary Figure 3). KEGG pathway enrichment showed that 130 pathways were enriched in TNNI2 vs. NC, of which the top 30 enriched pathways are displayed (Supplementary Table 7). Similarly, 30 of 108 pathways in ACTA1 vs. NC are shown in Supplementary Table 8. All the 22 pathways in TNNI2-ACTA1 V1 vs. NC are shown in Supplementary Table 9. Compared with groups TNNI2 and ACTA1, five specific pathways are shown in Table 2.

**Table 2:** Specific pathways enriched in group TNNI2-ACTA1 V1 compared with group TNNI2 and group ACTA1.

**Pathway**	**KEGG pathway term level 1**	**KEGG pathway term level 2**
Viral protein interaction with cytokine and cytokine receptor	Environmental information processing	Signaling molecules and interaction
Cytokine–cytokine receptor interaction	Environmental information processing	Signaling molecules and interaction
Chemokine signaling pathway	Organismal systems	Immune system
NF-κB signaling pathway	Environmental information processing	Environmental information processing
*Yersinia* infection	Human diseases	Infectious disease: bacterial

## Discussion

Gene fusion and fusion products (RNA and protein) are common phenomenon in cancer (13). For example, BCR-ABL caused by the translocation of chromosomes 9 and 22 is the target of Gleevec in the treatment of chronic myeloid leukemia (22). In the past few decades, chimeric RNAs were considered a special marker of cancer and were heavily sought after. These targets are being used as biomarkers or drug targets. But now, a variety of chimeric RNAs were found in non-cancer tissues. Scientists are beginning to believe that chimeric RNAs also have physiological effects in normal cells and tissues. With the improvement of high-throughput sequencing technology and the development of biological information, it provides an effective mean for the related research (11). More than 34,000 chimeric RNAs have been identified in multiple species. The production of chimeric RNAs has increased the diversity and complexity of transcriptomics and proteomics (23).

In this study, Star-Fusion and FusionMap were selected to analyze the RNA-seq data sets of the longissimus dorsi muscle and biceps femoris muscle of Min pigs. To minimize false discoveries due to library construction and sequencing errors, we focused exclusively on gene fusions detected by both programs. Based on the analysis, a total of 49 chimeric RNAs were jointly predicted by Star-Fusion and FusionMap. We randomly selected four candidate fusion transcripts and successfully validated one fusion and its eight transcript variants by RT-PCR and traditional Sanger sequencing. Candidate unique fusions had lower validation rates, presumably due to the difference in sample source, individuals, heterogeneity of tissues, and variable factors involved in cell culture (24). In addition, the differences of algorithm between Star-Fusion and FusionMap are an issue that cannot be ignored. Although the software for predicting chimeric RNAs have been improving constantly, the problems of false positives still exist and cause a lot of troubles for the verification of the predicted results.

As a muscle contraction regulator protein, the relationship of TNNI2 between structure and function has been recognized by the world (25). In recent years, other functions of the TNNI2 have gradually been discovered. Li et al. had reported that the TNNI2 can interact with nuclear receptors as a coactivator, which can be widely involved in physiological processes such as embryonic development, differentiation, and proliferation of cells (26). At present, the research for TNNI2 gene mainly focuses on a variety of congenital dyskinesias in humans. Mutations in related genes not only cause the patient's distal joints to bend but also include distal arthrogryposis syndrome (DA2A) (27) and Sheldon–Hall syndrome (DA2B) (28). Therefore, the normal expression of TNNI2 is important for the development of the skeletal muscle.

Actin is widespread in eukaryotes and is one of the most abundant proteins in eukaryotic cells (29). ACTA1 is widely involved in the assembly of muscle filaments, the development of skeletal muscle fibers, and the movement of cells and organelles (30). A number of researchers have reported that the mutation of the ACTA1 gene is closely related to a variety of human muscle diseases, such as congenital myopathy, disease in striated muscle, and nemaline myopathy (31–35).

For chimeric RNA, TNNI2-ACTA1 V1, the sixth exon of TNNI2 on chromosome 2 is fused with the fourth exon of ACTA1 on chromosome 14. The TNNI2-ACTA1 V2–V8 are formed in a similar way to TNNI2-ACTA1 V1. We speculate that chimeric RNA TNNI2-ACTA1 transcript variants were TS between precursor mRNAs transcribed from the two intact genes TNNI2 and ACTA1. As skeletal muscle developmental genes, TNNI2 and ACTA1 have played an important role in the development of skeletal muscle. Therefore, we suspect that chimeric RNAs formed by these two genes may play a role in the development of skeletal muscle.

To explore these issues, we designed and constructed the plasmids, pCMV-HA-TNNI2-ACTA1 V1–V8, pCMV-HA-TNNI2, and pCMV-HA-ACTA1, to enhance these mRNA expression levels in PSCs. The CCK-8 and EdU staining results showed that compared with the NC group, the TNNI2-ACTA1 V1 group inhibits the proliferation of PSCs. Interestingly, there is no obvious effect on PSC proliferation of the TNNI2 group, ACTA1 group, and transcript variants V2–V8. This result indicates that the function of TNNI2-ACTA1 V1 is completely different from the parental genes in terms of cell proliferation.

The cell cycle is the basic process of cell life activities and divided into four phases: gap 1 (G1), DNA synthesis (S), gap 2 (G2), and mitosis (M) (36). The operation of the cell cycle is orderly, and this strict operation is inseparable from the orderly expression of related regulatory genes, such as cyclins, cyclin-dependent kinases (CDKs), and CDK inhibitors (37). After transfection of the pCMV-HA-TNNI2-ACTA1 V1 vector into the PSCs, the proportion of G1 phase cells is increased by 8.8% (*p* < 0.001), the proportion of S phase cells is decreased by 4.76% (*p* < 0.01), and the proportion of G2/M phase cells is decreased by 4.03% (*p* < 0.05). Our results showed that the cell cycle was arrested in the G1 phase in TNNI2-ACTA1 V1 groups. The G1/S acts as a molecular switch, controlling the cell to continue dividing or enter a static state. The expression levels of cyclinDs (cyclinD1, cyclinD2, and cyclinD3) are essential for cells to enter S phase from G1 (38). We detected G1/S phase–related genes and found that the expression level of cyclinD1 was decreased in all three groups (*p* < 0.05). Interestingly, although the expression levels of cyclinD1 in the TNNI2 and ACTA1 groups were also decreased; the expression level of cyclinD3 was increased (*p* < 0.01). Previous studies have shown that the cyclinD proteins have high homology, and they can compensate each other, as long as one of them has a sufficient level of expression (39). This may be the reason why the TNNI2 and ACTA1 groups have no inhibitory effect on cell proliferation. Chimeric RNA TNNI2-ACTA1 V1 can decrease the expression level of cyclinD1 but cannot increase the expression level of other cyclinDs to compensate such as TNNI2 and ACTA1 groups. It caused the cell cycle to be arrested in the G1 phase.

To further explore the role of chimeric RNA TNNI2-ACTA1 V1 on cell proliferation, we performed transcriptome analysis by NGS. We observed that many of the pathways and biological processes in transfection TNNI2 or ACTA1 PSCs were significantly upregulated or downregulated. In particular, those pathways, which were mainly involved in interactions of signal molecules, signal transduction, and cell growth, were enriched. For instance, there are PI3K-Akt signaling pathway, MAPK signaling pathway, EGFR tyrosine kinase inhibitor resistance, and FoxO signaling pathway. As we all know, cell proliferation is a complex biological process, which involves complex interactions by gene regulatory network. These factors are interacting in cells to make cells relatively stable. This may be the reason why the result of cell proliferation is no change after transfection of the TNNI2 and ACTA1. Transcriptome analysis results show that, compared with the parental gene groups, three specific genes were enriched in the TNNI2-ACTA1 V1 group (NCOA3, Radixin, and DDR2). The results of previous studies have shown that NCOA3 plays a notable role in physiological and pathological functions, including somatic cell growth, sexual maturity, energy metabolism, female reproductive function, and tumorigenesis (40, 41). Karmakar's research results showed that depletion of NCOA3 modestly decreased cyclinD1 expression (42). Ezrin, radixin, and moesin play a significant role in regulating cells' life activities, such as cell growth, movement, migration, mitosis, and signal transduction (43). Discoidin domain receptors are a subfamily of the receptor tyrosine kinases (44). Furthermore, Zhang's research results revealed for the first time that DDR2 controls the expression of osteogenic markers by regulating the activation of the main transcription factor Runx2 and plays an important role in osteoblast differentiation and cartilage maturation (45). These research results are consistent with our study that the three genes may be the key to TNNI2-ACTA1 regulating cell proliferation.

Compared with the TNNI2 and ACTA1 groups, although none of the enriched-specific pathways are in the TNNI2-ACTA1 V1 group in pathways that related growth and development, the reason for this result may be that there are too few genes enriched in the pathway. Transcriptome analysis also found that the gene expression levels of cyclinDs and cyclinEs have the same trend as our experimental results, indicating that our experimental results are credible.

In summary, eight chimeric RNA TNNI2-ACTA1 transcript variants were successfully identified by analysis of high-throughput sequencing data sets. Based on the function of TNNI2 and ACTA1, we concentrated on the roles of TNNI2-ACTA1 V1–V8 in PSC proliferation. The CCK-8 assay and EdU staining results showed that TNNI2-ACTA1 V1 can inhibit the PSC proliferation. PSCs were arrested in the G1 phase by transfection pCMV-HA-TNNI2-ACTA1 V1 and the expression level of cyclinD1 was decreased. Interestingly, TNNI2-ACTA1 V2–V8 and parental genes TNNI2 and ACTA1 do not have this function. The mutual compensation between cyclinD1 and cyclinD3 may be the reason why TNNI2 and ACTA1 do not inhibit cell proliferation. The TNNI2-ACTA1 V1 may regulate cell growth via one or more of the expression levels of NCOA3, Radixin, and DDR2. Nevertheless, the mechanism of TNNI2-ACTA1 V1 through the three differential genes for regulating cell growth needed to be further explored. The functions of TNNI2-ACTA1 V2–V8 wait for further studies. In short, our research explores the role of chimeric RNAs in normal tissues and provides a theoretical basis for the mechanism of muscle growth.

## Data Availability Statement

The datasets presented in this study can be found in online repositories. The names of the repository/repositories and accession number(s) can be found here: NCBI SRA; PRJNA748219.

## Author Contributions

DL and XY came up with and designed the study. DL performed the experiments and wrote the manuscript. DL, JX, ZY, XZ, JL, and WH performed the statistical analysis. XY revised the manuscript. All authors contributed to the article and approved the submitted version.

## Funding

This research was supported by the National Natural Science Foundation of China (31741114).

## Conflict of Interest

The authors declare that the research was conducted in the absence of any commercial or financial relationships that could be construed as a potential conflict of interest.

## Publisher's Note

All claims expressed in this article are solely those of the authors and do not necessarily represent those of their affiliated organizations, or those of the publisher, the editors and the reviewers. Any product that may be evaluated in this article, or claim that may be made by its manufacturer, is not guaranteed or endorsed by the publisher.
