# Supplementary material for: Identification of Chimeric RNAs in Pig Skeletal Muscle and Transcriptomic Analysis of Chimeric RNA TNNI2-ACTA1 V1
Source: Front Vet Sci. 2021 Oct 27;8:742593. doi: 10.3389/fvets.2021.742593 (PMC8578878; doi:10.3389/fvets.2021.742593)
Supplement: Supplementary file 1 [file Data_Sheet_1.docx]

Supplementary Material

# Supplementary Figures

1.1 The gene expression and expression density distribution in each sample

**Supplementary Figure 1.** (A) The histogram of gene expression. The X-axis represents an individual sample. The Y-axis represents the number of expressed genes. The color depth represents the expression level of genes; (B) The density plot of genes’ log10 (FPKM+1) distribution visualized by CummeRbund. The X-axis represents the log10 (FPKM+1) of all the genes. The Y-axis represents the genes’ distribution density. The twelve groups were shown indifferent colors.

1.2 The volcano plot for differentially expressed genes in each group

**Supplementary Figure 2.** Volcano plot for differentially expressed genes (DEGs). The X-axis represents the difference multiple after log2 conversion, the Y-axis represents the significant value after-log10 conversion. Red represents up-regulated DEGs. Green represents down-regulated DEGs. Grey represents no-DEGs. (A) DEGs of Group TNNI2; (B) DEGs of Group ACTA1; (C) DEGs of Group TNNI2-ACTA1 V1;

1.3 The KEGG analysis for DEGs

**Supplementary Figure 3.** The column diagrams for KEGG analysis of DEGs. The X-axis represents the numbers of DEGs. The Y-axis represents the functions of pathways. Each color respective the fairish biological process. (A) DEGs of Group TNNI2; (B) DEGs of Group ACTA1; (C) DEGs of Group TNNI2-ACTA1 V1.

# Supplementary Tables

2.1 Identification of chimeric RNA by RT-PCR and the primer sequences were shown in Supplementary Table 1.

Supplementary Table 1. Primer sequences for Chimeric RNAs identification

| Chimeric RNA | Primer Sequences (5'-3') |
| --- | --- |
| ALDOA-ACTA1 | F: CGAGAACACCGAGGAGAACCG |
|  | R: TTAGAAGCATTTGCGGTGGACG |
| MYH1-ACTA1 | F: CTCCTTCAGGTCCTCTTGGC |
|  | R: TTAGAAGCATTTGCGGTGGACG |
| TNNT3-ACTA1 | F: ATGTCGGACGAGGAAGTAGAACAC |
|  | R: TTAGAAGCATTTGCGGTGGACG |
| TNNI2-ACTA1 | F: ATGGGGGATGAGGAGAAGCGC |
|  | R: TTAGAAGCATTTGCGGTGGACG |

2.2 The primer sequences for expression plasmid constructions were shown in Supplementary Table 2.

Supplementary Table 2. Primer sequences for expression plasmid constructions

| Gene | Primer Sequences (5'-3') | Production length (bp) |
| --- | --- | --- |
| TNNI2 | F:GAATTCCGATGGGGGATGAGGAGAAGC | 549 |
|  | R:GGTACCCTAGGACTCCGTCTCGAACA |  |
| ACTA1 | F:GAATTCCGATGTGTGACGAAGACGAG | 1134 |
|  | R:GGTACCTTAGAAGCATTTGCGG |  |
| TNNI2-ACTA1-V1 | F: GAATTCCGATGGGGGATGAGGAGAAGC | 870 |
| TNNI2-ACTA1-V2 |  | 819 |
| TNNI2-ACTA1-V3 |  | 525 |
| TNNI2-ACTA1-V4 |  | 720 |
| TNNI2-ACTA1-V5 | R: GGTACCTTAGAAGCATTTGCGG | 423 |
| TNNI2-ACTA1-V6 |  | 234 |
| TNNI2-ACTA1-V7 |  | 369 |
| TNNI2-ACTA1-V8 |  | 414 |

2.3 The primer sequences for detect the changes of cell cycle related genes were shown in Supplementary Table 3.

Supplementary Table 3. Primer sequences for the cell cycle related genes

| Gene | Primer Sequences (5'-3') | Production length (bp) |
| --- | --- | --- |
| CCND1 | F: TGCATCTACACCGACAACTCCA | 222 |
|  | R: GTTGGAAATGAACTTCACGTCTGT |  |
| CCND2 | F: CTGGATGCTGGAGGTCTGTGAG | 218 |
|  | R: GAGGCTTGATGGAGTTGTCGGT |  |
| CCND3 | F: TGGATGCTGGAGGTGTGTGAGG | 220 |
|  | R: GGCGGGGAGAGACAGAGTGGTC |  |
| CCNE1 | F: TGCCTTGTATCATTTCTCTT | 302 |
|  | R: GCTTCTTACTGCTCGGTG |  |
| CCNE2 | F: ATTCCCCTCAAGAAGCCCAAAT | 156 |
|  | R: TAATGCAAGGACTGATCCCCCC |  |
| GAPDH | F: CGGCACAGTCAAGGCGGAGAAC | 212 |
|  | R: CATCGGCAGAAGGGGCAGAGAT |  |

2.4 Real-time PCR was used to validate the RNA-Seq data. The primer sequences for this assay were shown in Supplementary Table 4.

Supplementary Table 4. Primer sequences for the real-time PCR to validate the RNA-Seq data

| Gene | Primer Sequences (5'-3') | Production length (bp) |
| --- | --- | --- |
| KLF7 | F: ATTAACGCCCCCGTCGTCCCCT | 208 |
|  | R: TCCTTGTTCGCTGTCGCTCTGT |  |
| CLK | F: GATGAACGCACACTGAAAAACA | 148 |
|  | R: CACAAGGCTGAGACCAACCTAA |  |
| ENO3 | F: CTGCTGGAAAAGAAACTAAGTG | 174 |
|  | R: AATGTGACGGTAGAGTGGGACC |  |
| PTGES | F: GGATGCTCAGAGACACGGAGGC | 114 |
|  | R: CCCAGGAACAGGAAGGGGTAAA |  |
| EIF4A2 | F: CATCTATGCTTACGGTTTTGAG | 114 |
|  | R: GCTGTCTTGCCAGTACCTGACT |  |
| WNT16 | F: ACCACGGGAAAAGAGAGCAAAG | 100 |
|  | R: ACCGGCAGTCTAGTGACATCAG |  |
| PRRX1 | F: GAGAAAGCAGCGGAGAAACAGG | 234 |
|  | R: GAGTAGGATTTGAGGAGGGAAG |  |
| BACE2 | F: CCTCCGCAGAAGCTGCAGATAC | 190 |
|  | R: TGACAACGTCCTCCCCAACAAG |  |
| KLF12 | F: GGTACATCCGTCCCCAGTGTCC | 228 |
|  | R: TTCTCTCCTGTGTGCGTCCTTC |  |
| NCOA3 | F: GAGGGTGTGGGGACTTCTCTTT | 120 |
|  | R: GGGACTCTTGGAATCCTGACTG |  |
| DDR2 | F: GCCATCATTGTCATCATCCTCT | 284 |
|  | R: CCCACTATTTCATCTTCACCTG |  |
| PRG4 | F: GACTACTTTGCACAATGGGACA | 240 |
|  | R: TTGGAAATCAGTTTGGGATACC |  |
| CAST | F: CAAGTCAGGAGAACAGAAAGGA | 126 |
|  | R: GTTGAAGCAGAGGAAGGCGATA |  |
| MEF2A | F: AGTTCACTTGTGTCCCCGTCTT | 130 |
|  | R: TGCCTGTACTTGGTGGTCTCTG |  |
| GAPDH | F: CGGCACAGTCAAGGCGGAGAAC | 212 |
|  | R: CATCGGCAGAAGGGGCAGAGAT |  |

2.5 These 49 Chimeric RNAs were jointly predicted by Star-Fusion and Fusionmap. The specific information was shown in Supplementary Table 5.

Supplementary Table 5. The specific information of Chimeric RNAs were jointly predicted by Star-Fusion and Fusionmap

| Chimeric RNA | Left Gene | Right Gene | Type | Annotation |
| --- | --- | --- | --- | --- |
| CKM--ACTA1 | CKM | ACTA1 | Inter | NC_010448.4--NC_010456.5 |
| TPM2--ACTA1 | TPM2 | ACTA1 | Inter | NC_010443.5--NC_010456.5 |
| MYH1--ACTA1 | MYH1 | ACTA1 | Inter | NC_010454.4--NC_010456.5 |
| CKM--TNNC2 | CKM | TNNC2 | Inter | NC_010448.4--NC_010459.5 |
| PYGM--ACTA1 | PYGM | ACTA1 | Inter | NC_010444.4--NC_010456.5 |
| ACTA1--TTN | ACTA1 | TTN | Inter | NC_010456.5--NC_010457.5 |
| CKM--MYH2 | CKM | MYH2 | Inter | NC_010448.4--NC_010454.4 |
| MYLPF--ACTA1 | MYLPF | ACTA1 | Inter | NC_010445.4--NC_010456.5 |
| TNNI2--MYH1 | TNNI2 | MYH1 | Inter | NC_010444.4--NC_010454.4 |
| ACTN3--CKM | ACTN3 | CKM | Inter | NC_010444.4--NC_010448.4 |
| GAPDH--ACTA1 | GAPDH | ACTA1 | Inter | NC_010447.5--NC_010456.5 |
| CKM--MYH1 | CKM | MYH1 | Inter | NC_010448.4--NC_010454.4 |
| ACTN3--ACTA1 | ACTN3 | ACTA1 | Inter | NC_010444.4--NC_010456.5 |
| TPM1--MYH1 | TPM1 | MYH1 | Inter | NC_010443.5--NC_010454.4 |
| TNNT3--ENO3 | TNNT3 | ENO3 | Inter | NC_010444.4--NC_010454.4 |
| ALDOA--ENO3 | ALDOA | ENO3 | Inter | NC_010445.4--NC_010454.4 |
| ACTN3--MYH4 | ACTN3 | MYH4 | Inter | NC_010444.4--NC_010454.4 |
| MYLPF--ATP2A1 | MYLPF | ATP2A1 | Intra | NC_010445.4 |
| TPM1--TNNI2 | TPM1 | TNNI2 | Inter | NC_010443.5--NC_010444.4 |
| GAPDH--MYH4 | GAPDH | MYH4 | Inter | NC_010447.5--NC_010454.4 |
| TNNT3--ACTA1 | TNNT3 | ACTA1 | Inter | NC_010444.4--NC_010456.5 |
| ALDOA--ACTA1 | ALDOA | ACTA1 | Inter | NC_010445.4--NC_010456.5 |
| TNNT3--CKM | TNNT3 | CKM | Inter | NC_010444.4--NC_010448.4 |
| TNNT3--TNNC2 | TNNT3 | TNNC2 | Inter | NC_010444.4--NC_010459.5 |
| TNNI2--ALDOA | TNNI2 | ALDOA | Inter | NC_010444.4--NC_010445.4 |
| ATP2A1--ACTA1 | ATP2A1 | ACTA1 | Inter | NC_010445.4--NC_010456.5 |
| TNNI2--ACTA1 | TNNI2 | ACTA1 | Inter | NC_010444.4--NC_010456.5 |
| GAPDH--CKM | GAPDH | CKM | Inter | NC_010447.5--NC_010448.4 |
| ALDOA--ATP2A1 | ALDOA | ATP2A1 | Intra | NC_010445.4 |
| GAPDH--MYH1 | GAPDH | MYH1 | Inter | NC_010447.5--NC_010454.4 |
| ENO3--MYH4 | ENO3 | MYH4 | Intra | NC_010454.4 |
| PYGM--GAPDH | PYGM | GAPDH | Inter | NC_010444.4--NC_010447.5 |
| CKM--TPT1 | CKM | TPT1 | Inter | NC_010448.4--NC_010453.5 |
| TNNI2--PYGM | TNNI2 | PYGM | Intra | NC_010444.4 |
| MYH4--ACTA1 | MYH4 | ACTA1 | Inter | NC_010454.4--NC_010456.5 |
| MYH1--TTN | MYH1 | TTN | Inter | NC_010454.4--NC_010457.5 |
| CKM--ENO3 | CKM | ENO3 | Inter | NC_010448.4--NC_010454.4 |
| YBX3--ACTA1 | YBX3 | ACTA1 | Inter | NC_010447.5--NC_010456.5 |
| TPM2--TNNT3 | TPM2 | TNNT3 | Inter | NC_010443.5--NC_010444.4 |
| TNNI2--MYH7 | TNNI2 | MYH7 | Inter | NC_010444.4--NC_010449.5 |
| TNNT3--PYGM | TNNT3 | PYGM | Intra | NC_010444.4 |
| TNNI2--CKM | TNNI2 | CKM | Inter | NC_010444.4--NC_010448.4 |
| TPM1--TNNT3 | TPM1 | TNNT3 | Inter | NC_010443.5--NC_010444.4 |
| RYR1--ACTA1 | RYR1 | ACTA1 | Inter | NC_010448.4--NC_010456.5 |
| MYLPF--ALDOA | MYLPF | ALDOA | Intra | NC_010445.4 |
| MYBPC2--TNNC2 | MYBPC2 | TNNC2 | Inter | NC_010448.4--NC_010459.5 |
| ALDOA--EEF1A2 | ALDOA | EEF1A2 | Inter | NC_010445.4--NC_010459.5 |
| TPM1--MYBPC2 | TPM1 | MYBPC2 | Inter | NC_010443.5--NC_010448.4 |
| ALDOA--LOC110257453 | ALDOA | LOC110257453 | Inter | NC_010445.4--NC_010459.5 |

* Inter: Inter-chromosomal; Intra: Intra-chromosomal.

2.6 The clean reads quality metrics were shown in Supplementary Table 6.

Supplementary Table 6. Statistics of the mapping of sequencing reads to the reference genome Sus scrofa 11.1

| Group | Individual | Clean Reads(M) | % Uniquely Mapping | Q20 (%) |
| --- | --- | --- | --- | --- |
| TNNI2 | TNNI2_1 | 23.91 | 91.42 | 98.17 |
|  | TNNI2_2 | 23.91 | 91.42 | 98.19 |
|  | TNNI2_3 | 23.91 | 91.19 | 98.25 |
| ACTA1 | ACTA1_1 | 23.91 | 91.37 | 98.16 |
|  | ACTA1_2 | 23.91 | 91.29 | 98.16 |
|  | ACTA1_3 | 23.9 | 91.31 | 98.18 |
| TNNI2-ACTA1 | TNNI2-ACTA1_1 | 23.91 | 91.36 | 98.42 |
|  | TNNI2-ACTA1_2 | 23.91 | 90.96 | 98.23 |
|  | TNNI2-ACTA1_3 | 23.91 | 90.94 | 98.2 |
| NC | NC_1 | 23.91 | 90.76 | 98.1 |
|  | NC_2 | 23.91 | 90.92 | 98.28 |
|  | NC_3 | 23.91 | 90.59 | 98.08 |

2.7 The top 30 pathways were enriched for group TNNI2 were shown in Supplementary Table 7.

Supplementary Table 7. Pathways enriched in Group TNNI2

| Pathway | p-Value | q-Value |
| --- | --- | --- |
| Focal adhesion | 2.40×10^-17^ | 7.92×10^-15^ |
| Ribosome biogenesis in eukaryotes | 7.51×10^-16^ | 1.24×10^-13^ |
| ECM-receptor interaction | 6.29×10^-13^ | 6.92×10^-11^ |
| Human papillomavirus infection | 5.09×10^-12^ | 4.20×10^-10^ |
| PI3K-Akt signaling pathway | 9.38×10^-9^ | 6.19×10^-7^ |
| Proteoglycans in cancer | 1.59×10^-8^ | 8.74×10^-7^ |
| Protein processing in endoplasmic reticulum | 2.85×10^-8^ | 1.34×10^-6^ |
| Pathways in cancer | 3.36×10^-8^ | 1.38×10^-6^ |
| Epstein-Barr virus infection | 4.64×10^-8^ | 1.61×10^-6^ |
| AGE-RAGE signaling pathway in diabetic complications | 4.87×10^-8^ | 1.61×10^-6^ |
| MAPK signaling pathway | 9.12×10^-8^ | 2.74×10^-6^ |
| Dilated cardiomyopathy (DCM) | 3.19×10^-7^ | 8.78×10^-6^ |
| Arrhythmogenic right ventricular cardiomyopathy (ARVC) | 4.65×10^-7^ | 1.18×10^-5^ |
| MicroRNAs in cancer | 5.04×10^-7^ | 1.19×10^-5^ |
| Cellular senescence | 7.01×10^-7^ | 1.54×10^-5^ |
| Hypertrophic cardiomyopathy (HCM) | 8.25×10^-7^ | 1.70×10^-5^ |
| Transcriptional misregulation in cancer | 4.49×10^-6^ | 8.72×10^-5^ |
| Human cytomegalovirus infection | 7.32×10^-6^ | 1.34×10^-4^ |
| Rap1 signaling pathway | 8.50×10^-6^ | 1.48×10^-4^ |
| Shigellosis | 1.33×10^-5^ | 2.19×10^-4^ |
| Adherens junction | 1.49×10^-5^ | 2.34×10^-4^ |
| Regulation of actin cytoskeleton | 1.80×10^-5^ | 2.70×10^-4^ |
| Axon guidance | 2.04×10^-5^ | 2.93×10^-4^ |
| Human T-cell leukemia virus 1 infection | 3.17×10^-5^ | 4.36×10^-4^ |
| Melanoma | 3.63×10^-5^ | 4.80×10^-4^ |
| EGFR tyrosine kinase inhibitor resistance | 4.06×10^-5^ | 5.15×10^-4^ |
| Prostate cancer | 4.33×10^-5^ | 5.30×10^-4^ |
| Bladder cancer | 5.23×10^-5^ | 6.16×10^-4^ |
| Amoebiasis | 6.67×10^-5^ | 7.59×10^-4^ |
| Toxoplasmosis | 7.26×10^-5^ | 7.98×10^-4^ |

2.8 The top 30 pathways were enriched for group ACTA1 were shown in Supplementary Table 8.

Supplementary Table 8. Pathways enriched in Group ACTA1

| Pathway | p-Value | q-Value |
| --- | --- | --- |
| Ribosome | 9.11×10^-14^ | 3.03×10^-11^ |
| Focal adhesion | 1.12×10^-9^ | 1.86×10^-7^ |
| Protein processing in endoplasmic reticulum | 1.75×10^-8^ | 1.94×10^-6^ |
| Kaposi sarcoma-associated herpesvirus infection | 4.92×10^-8^ | 4.09×10^-6^ |
| Epstein-Barr virus infection | 9.60×10^-8^ | 6.39×10^-6^ |
| Proteoglycans in cancer | 1.96×10^-7^ | 1.09×10^-5^ |
| Viral carcinogenesis | 1.03×10^-6^ | 4.88×10^-5^ |
| Parkinson disease | 1.83×10^-6^ | 7.60×10^-5^ |
| Mitophagy - animal | 2.64×10^-6^ | 9.77×10^-5^ |
| Cellular senescence | 4.19×10^-6^ | 1.36×10^-4^ |
| Hypertrophic cardiomyopathy (HCM) | 4.67×10^-6^ | 1.36×10^-4^ |
| Dilated cardiomyopathy (DCM) | 4.91×10^-6^ | 1.36×10^-4^ |
| Shigellosis | 8.23×10^-6^ | 2.11×10^-4^ |
| Human papillomavirus infection | 9.50×10^-6^ | 2.26×10^-4^ |
| Fluid shear stress and atherosclerosis | 1.07×10^-5^ | 2.38×10^-4^ |
| HIF-1 signaling pathway | 1.22×10^-5^ | 2.54×10^-4^ |
| Alzheimer disease | 1.61×10^-5^ | 3.15×10^-4^ |
| MicroRNAs in cancer | 1.72×10^-5^ | 3.18×10^-4^ |
| TNF signaling pathway | 2.77×10^-5^ | 4.85×10^-4^ |
| Viral myocarditis | 3.54×10^-5^ | 5.80×10^-4^ |
| Oxidative phosphorylation | 3.78×10^-5^ | 5.80×10^-4^ |
| p53 signaling pathway | 3.83×10^-5^ | 5.80×10^-4^ |
| Human T-cell leukemia virus 1 infection | 4.63×10^-5^ | 6.71×10^-4^ |
| AGE-RAGE signaling pathway in diabetic complications | 5.21×10^-5^ | 7.23×10^-4^ |
| Bacterial invasion of epithelial cells | 7.91×10^-5^ | 1.05×10^-3^ |
| Non-alcoholic fatty liver disease (NAFLD) | 9.35×10^-5^ | 1.20×10^-3^ |
| EGFR tyrosine kinase inhibitor resistance | 1.01×10^-4^ | 1.25×10^-3^ |
| Endocytosis | 1.72×10^-4^ | 2.0×10^-3^ |
| Small cell lung cancer | 1.80×10^-4^ | 2.0×10^-3^ |
| ECM-receptor interaction | 1.86×10^-4^ | 2.0×10^-3^ |

2.9 All the pathways were enriched for group TNNI2-ACTA1 V1 were shown in Supplementary Table 9.

Supplementary Table 9. Pathways enriched in Group TNNI2-ACTA1 V1

| Pathway | p-Value | q-Value |
| --- | --- | --- |
| TNF signaling pathway | 1.59×10^-4^ | 1.28×10^-2^ |
| Legionellosis | 3.87×10^-4^ | 1.57×10^-2^ |
| Viral protein interaction with cytokine and cytokine receptor | 1.34×10^-3^ | 3.10×10^-2^ |
| IL-17 signaling pathway | 1.53×10^-3^ | 3.10×10^-2^ |
| Cytokine-cytokine receptor interaction | 4.31×10^-3^ | 7.0×10^-2^ |
| Influenza A | 7.30×10^-3^ | 9.86×10^-2^ |
| Chemokine signaling pathway | 1.07×10^-2^ | 1.20×10^-1^ |
| Viral carcinogenesis | 1.19×10^-2^ | 1.20×10^-1^ |
| Human T-cell leukemia virus 1 infection | 1.95×10^-2^ | 1.60×10^-1^ |
| Shigellosis | 2.0×10^-2^ | 1.60×10^-1^ |
| Rheumatoid arthritis | 2.38×10^-2^ | 1.60×10^-1^ |
| AGE-RAGE signaling pathway in diabetic complications | 2.84×10^-2^ | 1.60×10^-1^ |
| NF-kappa B signaling pathway | 2.89×10^-2^ | 1.60×10^-1^ |
| C-type lectin receptor signaling pathwaycardiomyopathy (ARVC) | 3.0×10^-2^ | 1.60×10^-1^ |
| Parathyroid hormone synthesis, secretion and action | 3.05×10^-2^ | 1.60×10^-1^ |
| Chagas disease (American trypanosomiasis) | 3.16×10^-2^ | 1.60×10^-1^ |
| Osteoclast differentiation | 4.21×10^-2^ | 1.81×10^-1^ |
| Yersinia infection | 4.21×10^-2^ | 1.81×10^-1^ |
| Fluid shear stress and atherosclerosis | 4.78×10^-2^ | 1.81×10^-1^ |
| Apoptosis | 4.85×10^-2^ | 1.81×10^-1^ |
| Apelin signaling pathway | 4.85×10^-2^ | 1.81×10^-1^ |
| Signaling pathways regulating pluripotency of stem cells | 4.91×10^-2^ | 1.81×10^-1^ |
