# Supplementary figures and images for: Identification of Chimeric RNAs in Pig Skeletal Muscle and Transcriptomic Analysis of Chimeric RNA TNNI2-ACTA1 V1
Source: Front Vet Sci. 2021 Oct 27;8:742593. doi: 10.3389/fvets.2021.742593 (PMC8578878; doi:10.3389/fvets.2021.742593)

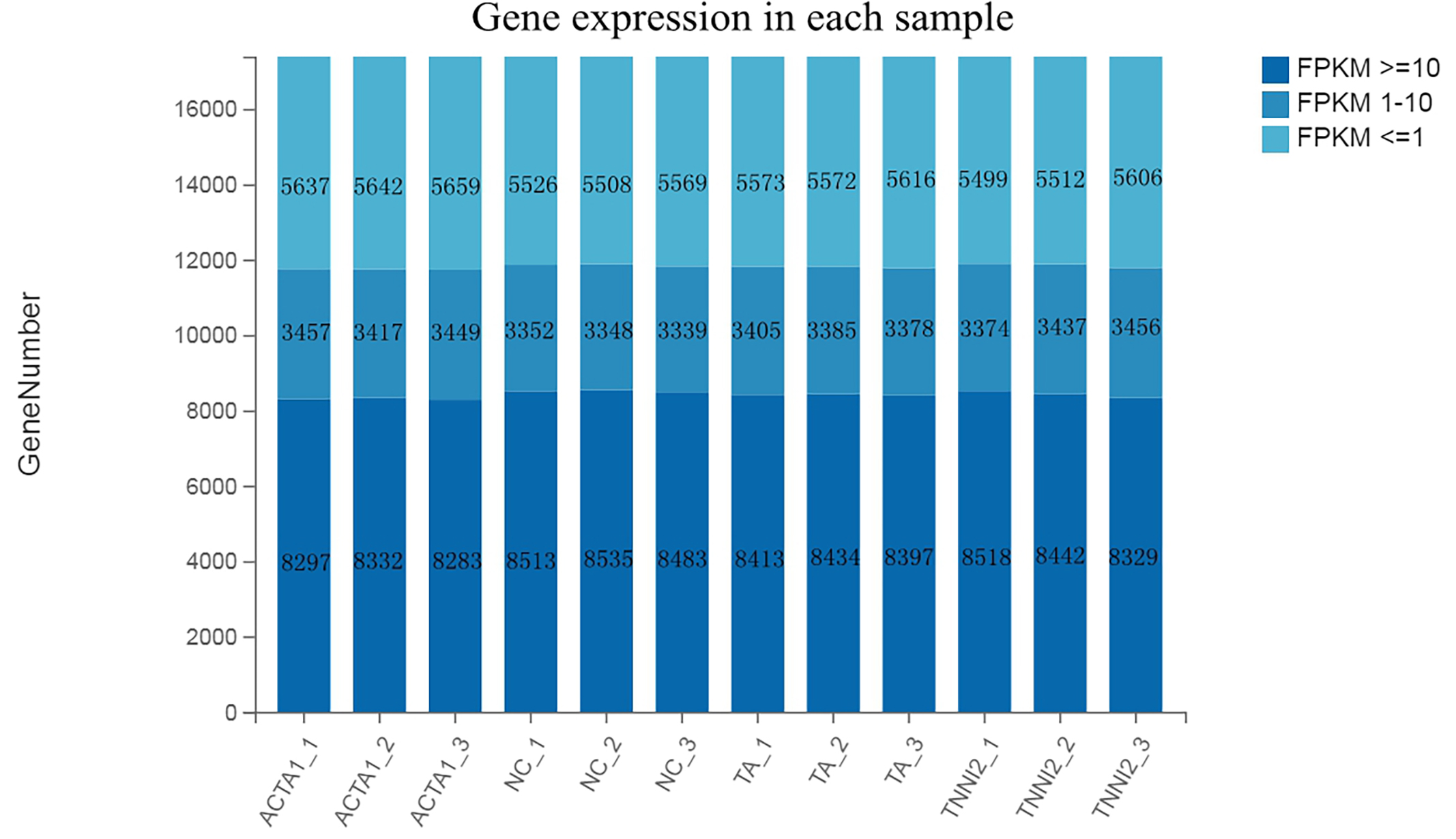

Supplement: Supplementary file 2 [file Image_1.TIF]

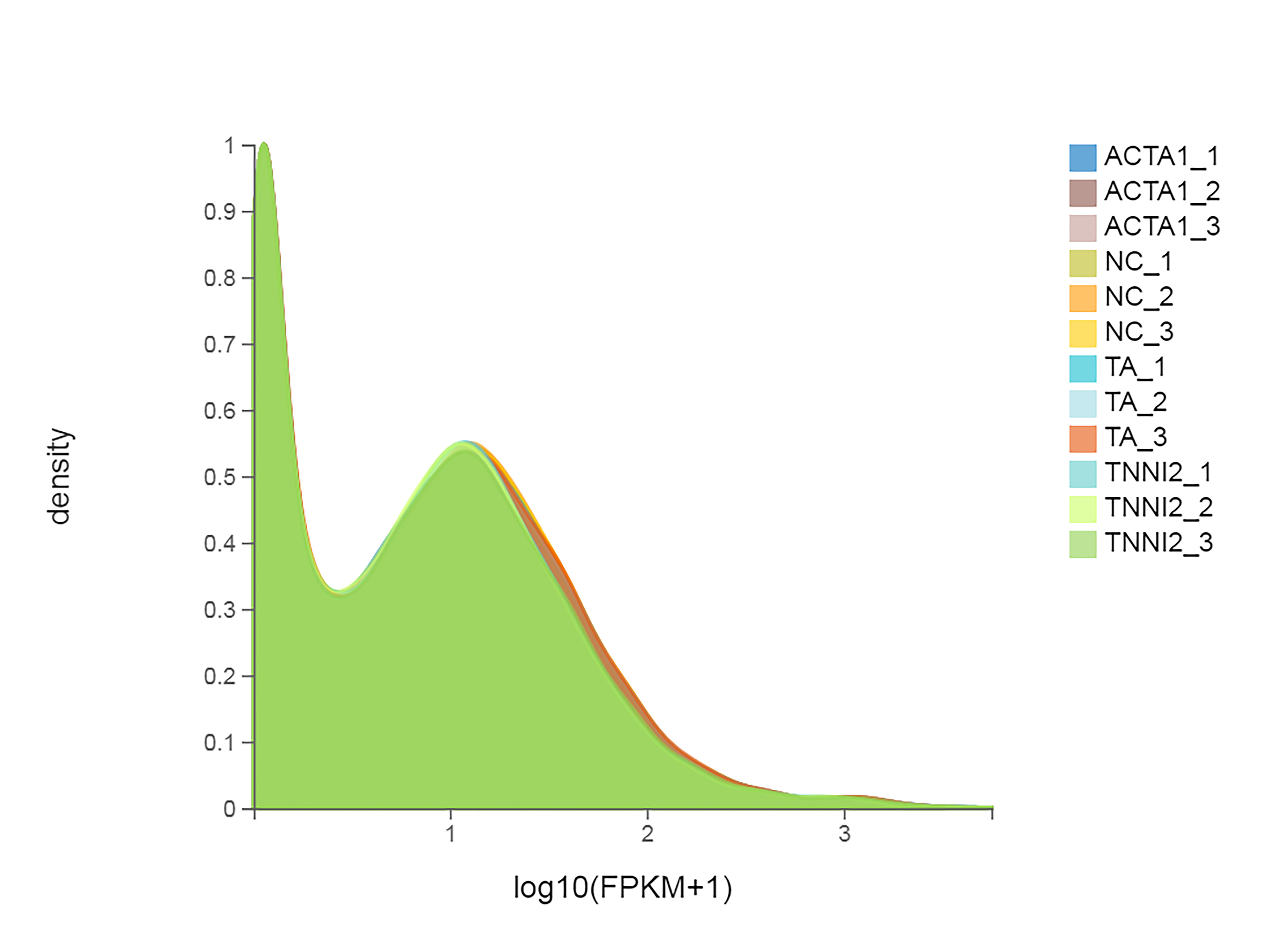

Supplement: Supplementary file 3 [file Image_2.TIF]

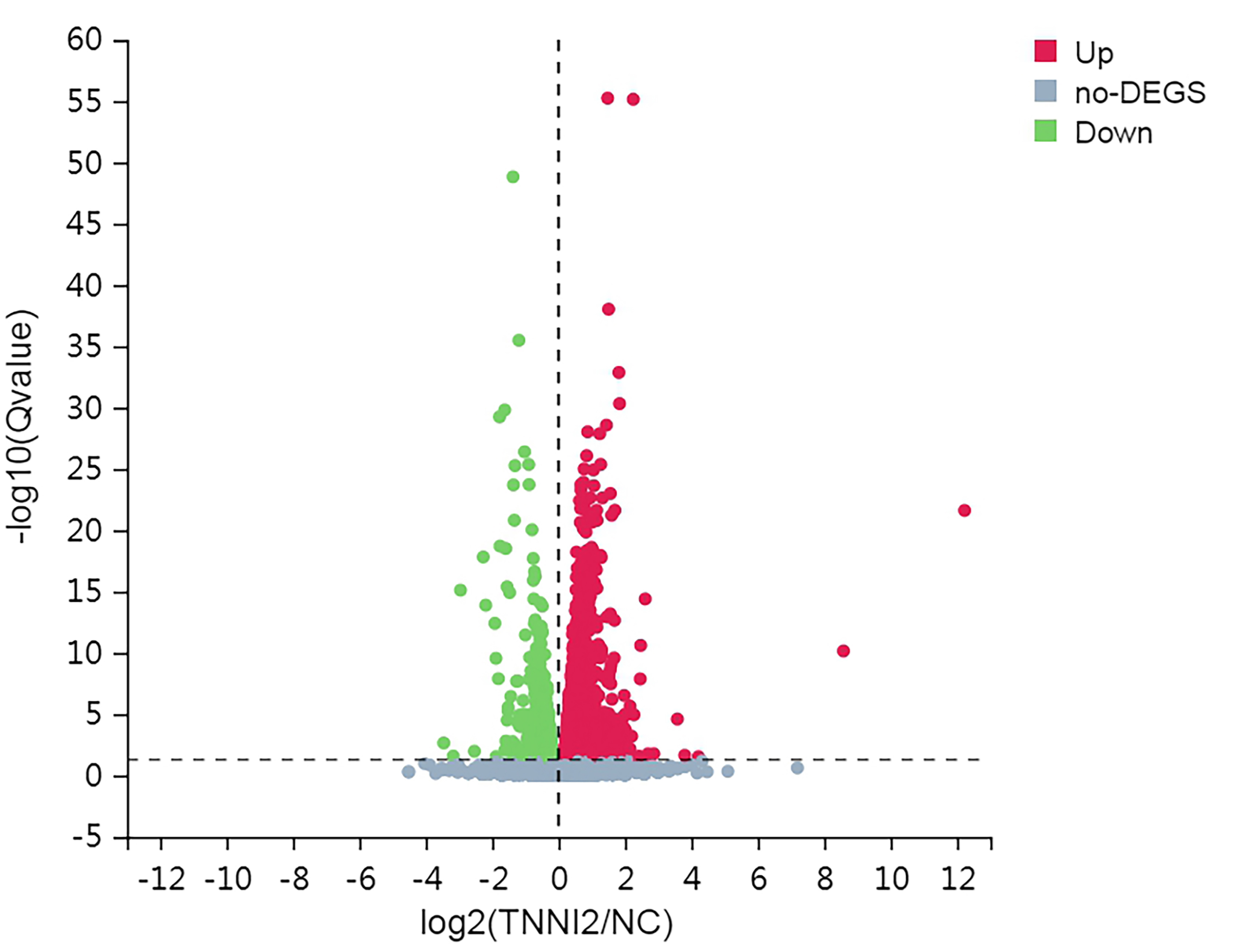

Supplement: Supplementary file 4 [file Image_3.TIF]

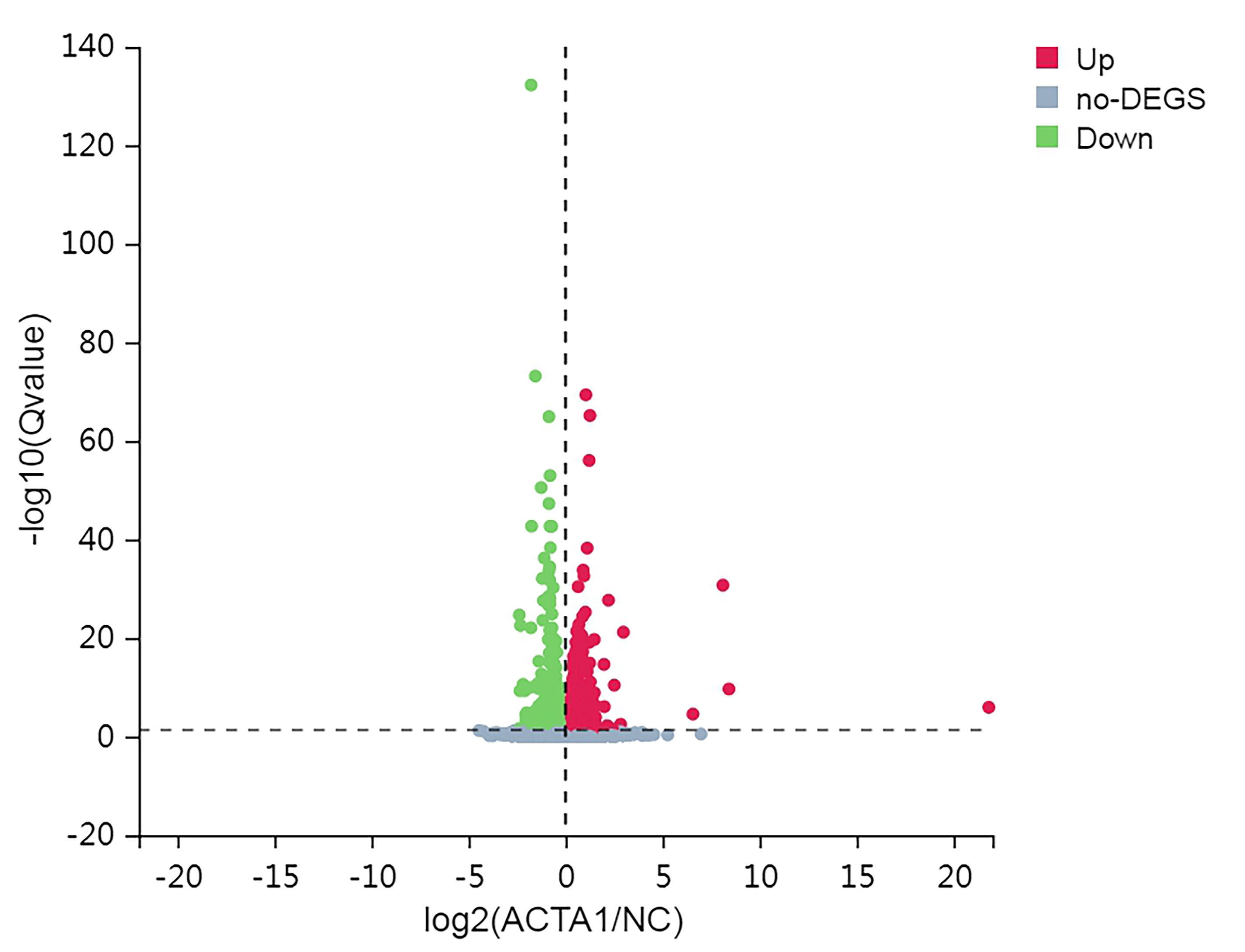

Supplement: Supplementary file 5 [file Image_4.TIF]

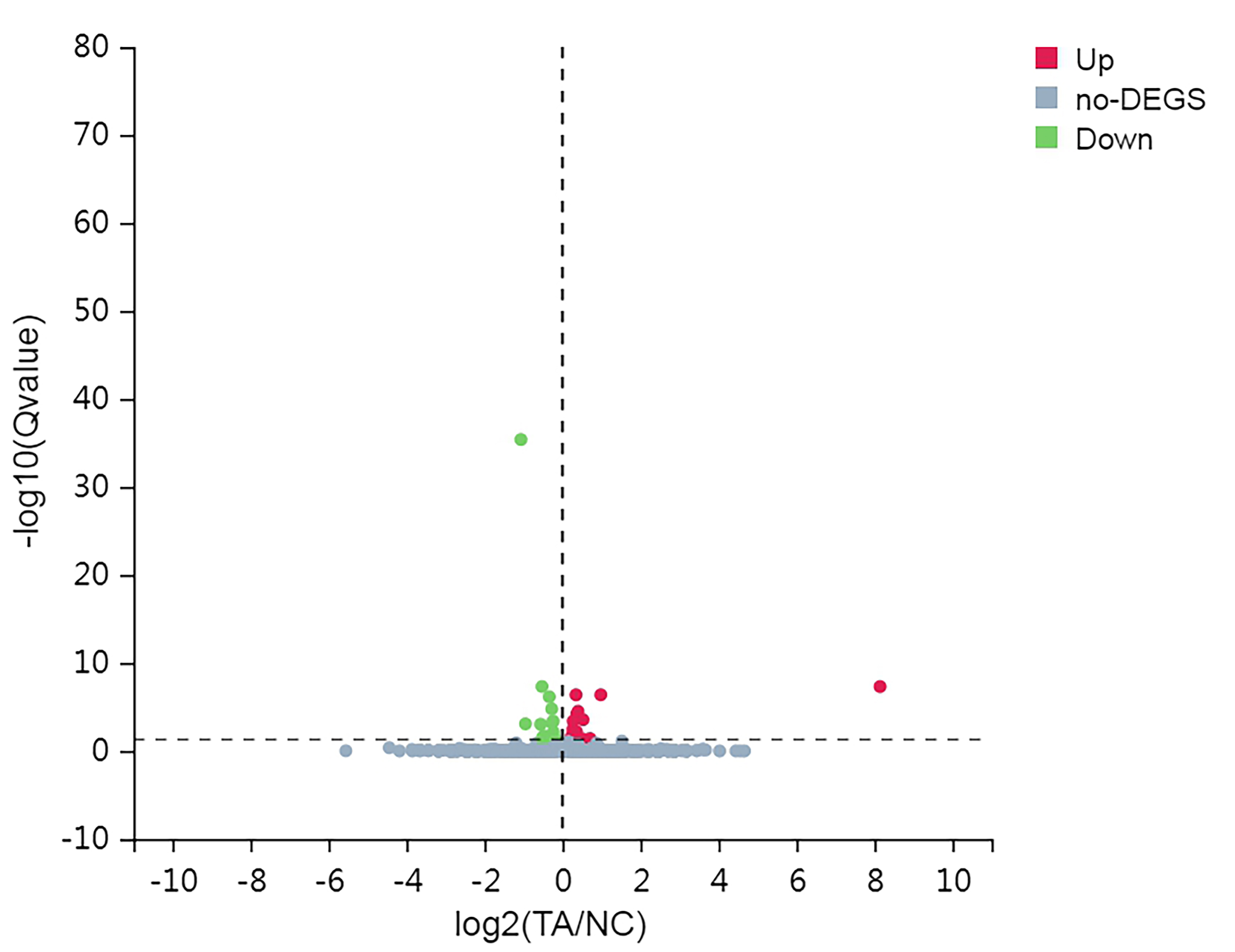

Supplement: Supplementary file 6 [file Image_5.TIF]

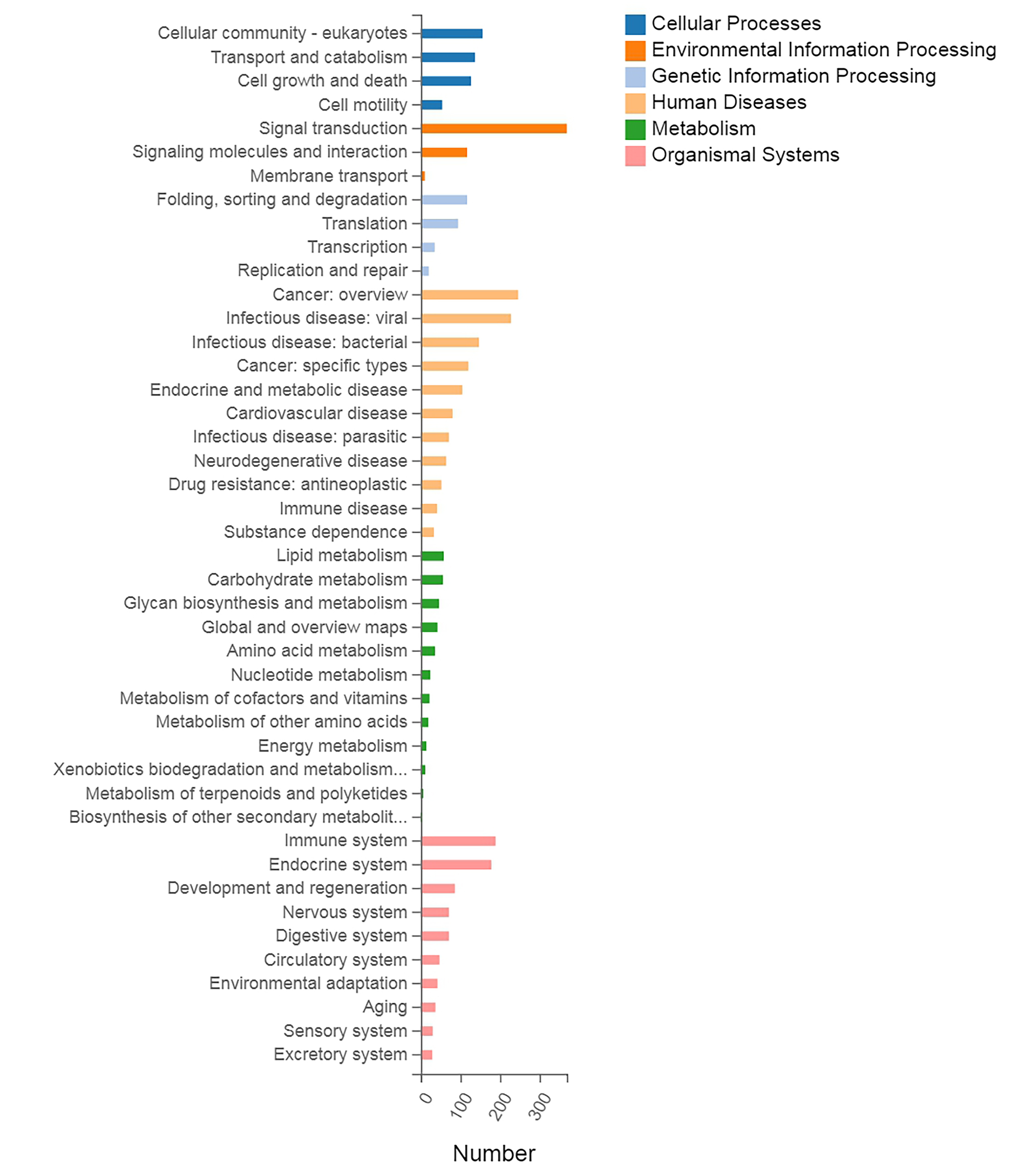

Supplement: Supplementary file 7 [file Image_6.TIF]

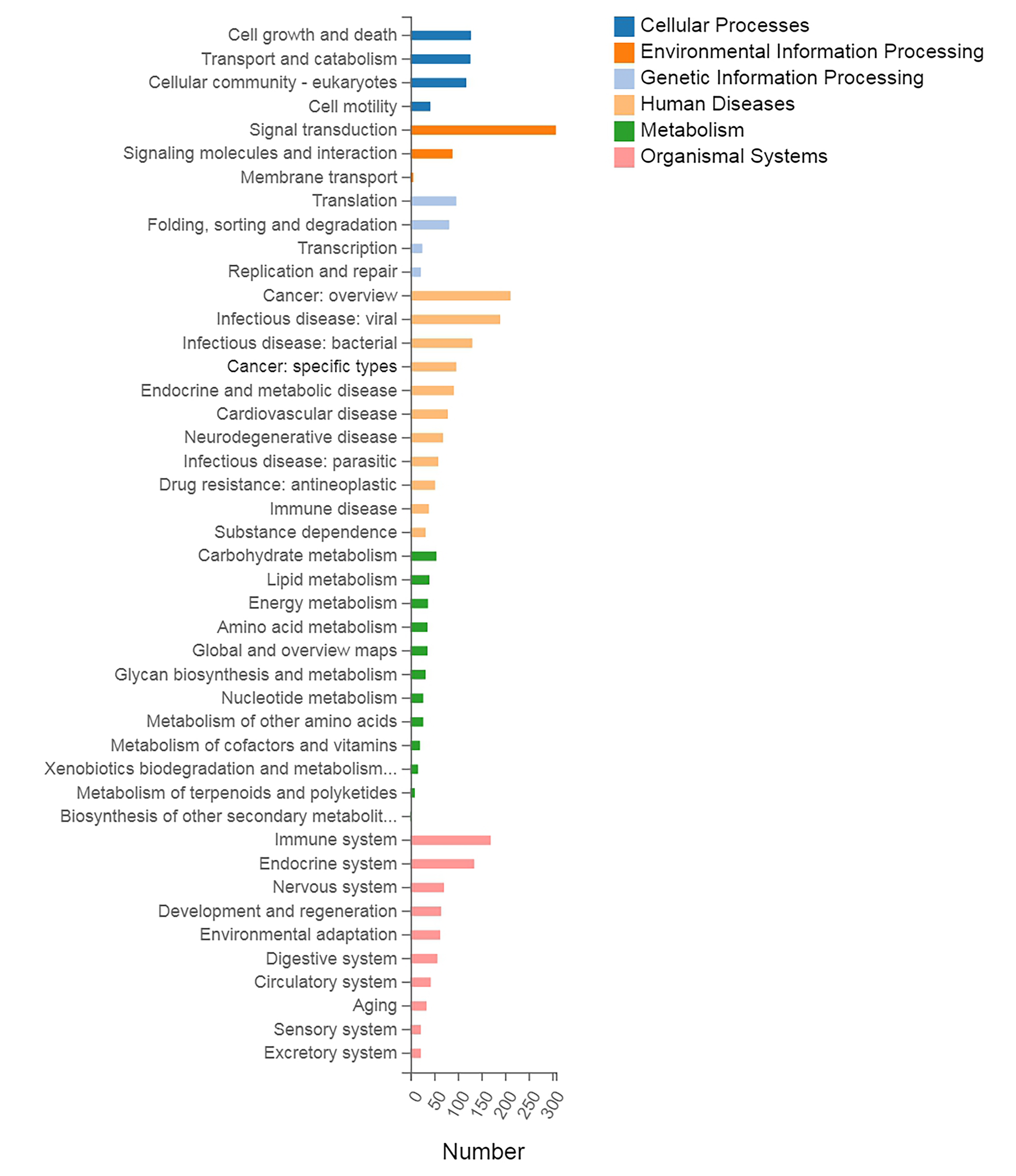

Supplement: Supplementary file 8 [file Image_7.TIF]

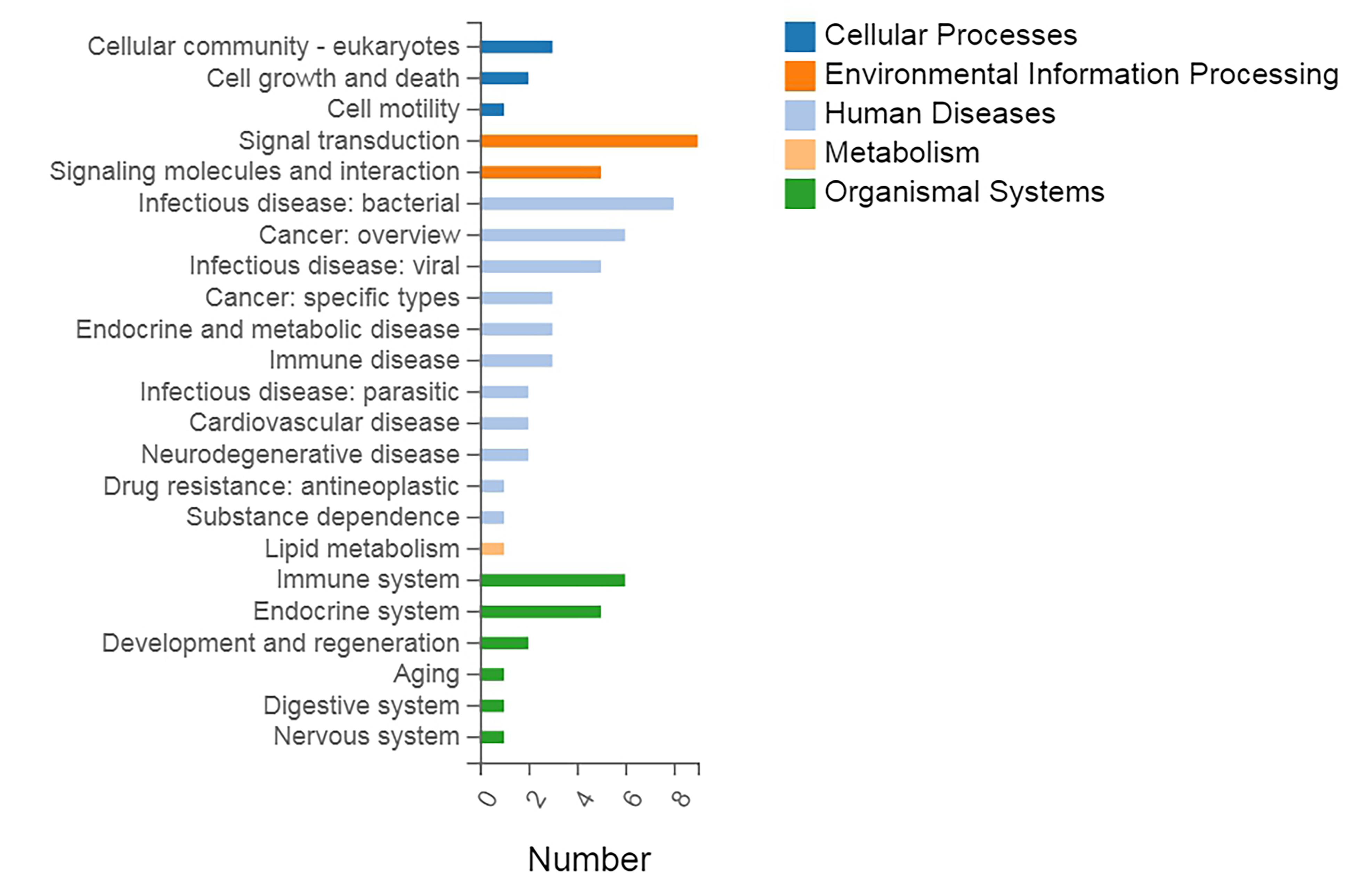

Supplement: Supplementary file 9 [file Image_8.TIF]
